# Biological and Catalytic Properties of Selenoproteins

**DOI:** 10.3390/ijms241210109

**Published:** 2023-06-14

**Authors:** Jean Chaudière

**Affiliations:** CBMN (CNRS, UMR 5248), University of Bordeaux, 33600 Pessac, France; jean.chaudiere@u-bordeaux.fr

**Keywords:** selenium, selenoenzyme, selenocysteine, catalytic mechanism, glutathione peroxidase, thioredoxin reductase, deiodinase, methionyl-sulfoxide reductase, glycine reductase, D-proline reductase, formate dehydrogenase, hydrogenase, antioxidant, redox regulation

## Abstract

Selenocysteine is a catalytic residue at the active site of all selenoenzymes in bacteria and mammals, and it is incorporated into the polypeptide backbone by a co-translational process that relies on the recoding of a UGA termination codon into a serine/selenocysteine codon. The best-characterized selenoproteins from mammalian species and bacteria are discussed with emphasis on their biological function and catalytic mechanisms. A total of 25 genes coding for selenoproteins have been identified in the genome of mammals. Unlike the selenoenzymes of anaerobic bacteria, most mammalian selenoenzymes work as antioxidants and as redox regulators of cell metabolism and functions. Selenoprotein P contains several selenocysteine residues and serves as a selenocysteine reservoir for other selenoproteins in mammals. Although extensively studied, glutathione peroxidases are incompletely understood in terms of local and time-dependent distribution, and regulatory functions. Selenoenzymes take advantage of the nucleophilic reactivity of the selenolate form of selenocysteine. It is used with peroxides and their by-products such as disulfides and sulfoxides, but also with iodine in iodinated phenolic substrates. This results in the formation of Se-X bonds (X = O, S, N, or I) from which a selenenylsulfide intermediate is invariably produced. The initial selenolate group is then recycled by thiol addition. In bacterial glycine reductase and D-proline reductase, an unusual catalytic rupture of selenium–carbon bonds is observed. The exchange of selenium for sulfur in selenoproteins, and information obtained from model reactions, suggest that a generic advantage of selenium compared with sulfur relies on faster kinetics and better reversibility of its oxidation reactions.

## 1. Introduction

**Figure 1:**
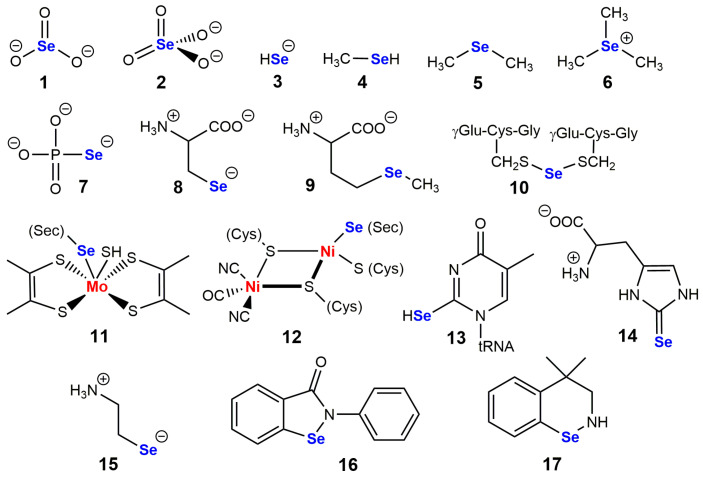
A few structures of selenium-containing molecules are of biological interest. (1) selenite; (2) selenate; (3) selenide; (4) methylselenol; (5) dimethylselenide; (6) trimethylselenonium; (7) selenophosphate; (8) selenocysteine; (9) selenomethionine; (10) glutathione selenotrisulfide; (11) dithiolene-molybdenum cluster of formate dehydrogenases; (12) nickel-sulfur cluster of bacterial hydrogenases; (13) 5-methyl-2-selenouridine in tRNA; (14) selenoneine, selenium analog of ergothioneine; (15) selenocysteamine; (16) and (17) are biomimetic catalysts (2-phenyl-1,2-benzisoselenazol-3-(2H)-one) and 4,4-dimethyl-benzisoselenazine, respectively known as ebselen and BXT-51072, which were tested in clinical development.

The development of selenium biochemistry was best summarized by Leopold Flohé, one of its major contributors [1]. Selenium is the 34th element in the periodic table, with an atomic mass of 78.96. It belongs to period 4 and group VIB of chalcogens. It was discovered in 1817 by Berzelius and Gahn, in the red-brown sediment of industrial tanks used for the preparation of sulfuric acid, where tellurium (from Latin *telluris* which means earth) had already been identified. Hence the choice of the name selenium, from the Greek *selene*, which meant “moon goddess”. As shown in Figure 1, a wide range of structures are observed in selenium-containing metabolites and essential biomolecules, as well as in synthetic selenium-containing mimics of selenoenzymes. Selenite SeO_3_^2−^ and selenate SeO_4_^2−^ are sources of selenium in drinking water, selenide HSe^−^ is an end-product of their biological reduction, and it can be enzymatically transformed into selenophosphate by ATP, or mono-, di- and tri-methylated by S-adenosyl methionine. Dimethylselenide Se(CH_3_)_2_ is excreted in breath, trimethylselenonium (CH_3_)_3_Se^+^ is excreted in urine, and selenophosphate is always used for specific incorporation of selenium into biomolecules. In most selenium-containing proteins, selenium is in the form of a selenocysteine residue. These are the “selenoproteins” discussed in this review, but there are also a few “selenium-binding proteins” in which selenium can be dissociated from the protein by thiol reagents, probably from persulfides of cysteine residues [2].

Selenium is an essential oligo-element in mammals including humans, but the window between deficiency and toxicity is rather narrow [3,4,5]. Signs of deficiency are observed for daily intakes of 18 µg or less, and signs of toxicity are observed above 400 µg. Depending on countries and organizations, the recommended daily intake varies between 30 and 75 µg in human adults. In humans, manifestations of severe deficiency include muscular and cardiovascular dysfunctions, osteochondropathy, and infertility. Moreover, large trials carried out among elderly persons have shown that a low selenium status is associated with a faster decline in cognitive functions [6]. The importance of selenoproteins in brain development, not only in aging, has been illustrated by reverse genetics [7]. A neurodevelopmental syndrome called progressive cerebello-cortical atrophy (PCCA) has also been shown to be caused by mutations in the selenocysteine synthase gene [8]. knock-out models have demonstrated that selenoproteins are specifically required in postmitotic neurons of the developing cerebellum [9].

Two endemic diseases which are associated with selenium deficiency have been largely studied in several Chinese regions, namely Keshan disease [10] which is severe cardiomyopathy, and Kashin–Beck disease [11] which is associated with degenerative lesions of joints and vertebral column. In deficient regions of China, Keshan disease was largely eradicated in the 1990s thanks to the addition of sodium selenite to table salt [12], and in Finland, the soil deficit in selenium has led to systematic addition of selenium in fertilization media [13].

In livestock, selenium deficiency also has severe consequences, and today, the selenium status of sheep, cows, pigs, and poultry is tightly controlled. Conversely, consequences of intoxication due to overload in bioavailable selenium—mainly selenite SeO_3_^2−^, selenate SeO_4_^2−^, selenomethionine and selenocysteine—i.e., selenium, which can be incorporated into natural selenoproteins or in place of sulfur in cysteine and methionine residues, may also be severe. The molecular origin of such toxic effects is mainly the nonspecific replacement of cysteine residues by selenocysteine residues whose selenol group may induce deleterious reactions in such modified proteins. By comparison, the replacement of methionine residues by the isosteric selenomethionine is also observed, but it has no or minor deleterious effect. A recent work performed on mice indicates however that for some proteins which are not selenoproteins, selenium provided in the form of selenocysteine or selenomethionine—not as inorganic salts—can lead to the selective replacement of cysteine or methionine by their selenium homologs at specific sites [14]. Such facultative selenation would not be random and may explain some physiological effects attributable to shifts in dietary selenium.

The administration of selenium at normally toxic doses may serve as an antidote to intoxication by heavy metals such as mercury or cadmium, most likely by forming stable complexes which facilitate their elimination [15,16]. The other side of the coin is that such heavy metals inhibit selenoenzymes, which is also the case for gold salts [17].

In the early 1970s, the identification of an essential atom of selenium incorporated in the form of selenocysteine at the active site of glutathione peroxidases Se-GPx [18,19] provided a first explanation for the essential role of selenium in mammals. Such enzymes catalyze the reduction of hydroperoxides [H_2_O_2_ and/or ROOH] by glutathione GSH, thereby playing a central role in the antioxidant system of mammals.

Selenomethionine is synthesized in plants where it is randomly incorporated into proteins at Met positions. Selenium of plant selenoproteins is exclusively in the form of selenomethionine which is therefore not specifically coded in the genomic sequence. The partial replacement of one or several methionine [AUG codon] by selenomethionine as a competitive ligand of the tRNA acceptor has structural and functional effects which may be negligible. In archaea, bacteria, and animals, selenium is on the contrary specifically incorporated into selenoproteins by co-translational insertion of selenocysteine. Selenocysteine is therefore the 21st amino acid which is coded in the genome, and which can be inserted in a growing polypeptide chain by means of a specific tRNA [20]. Its codon is UGA—normally read as a termination “opal” codon—which will be read as a “selenocysteine codon” in the context of a control sequence carried by the mRNA. The requirement for selenocysteine-containing proteins is most puzzling in insects. For example, *Drosophila melanogaster* and most other fruit flies possess three selenoprotein genes, but in *Drosophila willistoni*, selenocysteine has been replaced by cysteine and the organism has lost the capacity to synthesize selenocysteine [21].

## 2. Co-Translational Incorporation of Selenocysteine

This is a complex process that is only partially identical in prokaryotes and eukaryotes [20,22,23,24]. In eukaryotes, decoding UGA as a selenocysteine codon requires a specific tRNA, a dedicated elongation factor EFsec, and a selenocysteine insertion sequence SECIS which is a hairpin present in the untranslated 3’ region [3’-UTR] of all selenoprotein mRNAs. SECIS recruits all the UGA recoding tools which will impose the recognition of UGA as a “selenocysteine codon” instead of a termination codon. The specific tRNA that binds UGA is called tRNA(Ser)Sec because it is initially aminoacylated by serine which is then transformed into selenocysteyl-t-RNA in a co-translational process. tRNA(Ser)Sec is much longer (a hundred nucleotides) than other tRNAs and it has a markedly distinct 3D structure. This prevents its binding to the canonical transporter of tRNA known as EF1A and enables its processing by the EFsec elongation factor. Clustering of the SECIS elements in the 3’-UTR region upstream from the first UGA codon enables the controlled decoding of several UGA/selenocysteine codons for a protein containing several selenocysteines, such as selenoprotein P in mammals. By contrast, in prokaryotes, SECIS elements are not in the 3′-UTR region, but immediately downstream from the UGA codon, so that the SECIS structure is required for the decoding of each UGA/selenocysteine codon. One should underline that the efficiency of UGA recoding and selenoprotein m-RNA translation is typically low, with a majority of ribosomes failing to incorporate selenocysteine and reach the termination codon. It is outside the scope of this review to discuss all factors involved in this process, but the reader is referred to the review of Copeland and Howard [25] in which the role of accessory proteins and that of potential effectors of the No-Go Decay (NGD) mRNA pathway are discussed in detail.

The aminoacylation of tRNA-(Ser)Sec requires four enzymes instead of one, because the objective is not to load preformed selenocysteine, but to load serine (sidechain CH_2_OH, not CH_2_SeH) which will be transformed into selenocysteine on tRNA with an additional consumption of three ATP. These enzymes are seryl-tRNA synthetase, phosphoseryl-tRNA kinase, selenophosphate synthetase 2 which produces selenophosphate from selenide HSe^−^ and ATP, and Sec synthase.

This results in the following reactional sequence:

tRNA + Ser + ATP → tRNA-Ser + AMP + PPi Seryl-tRNA synthetase(1)

tRNA-Ser + ATP → tRNA-Ser-Ph + ADP Phosphoseryl-tRNA kinase(2)

HSe^−^ + ATP → AMP + PPi + H_2_SePO_3_^−^ Selenophosphate synthetase (3)

tRNA-Ser-Ph + H_2_SePO_3_^−^ → 2 Pi + tRNA-Sec Selenocysteine synthase (4)

Hydrogen selenide H_2_Se is produced in the cell by the reduction of selenite SeO_3_^2−^ through an intermediate selenodiglutathione GS-Se-SG, or from selenocysteine in the trans-selenation analog of the trans-sulfuration pathway.

**Figure 2:**
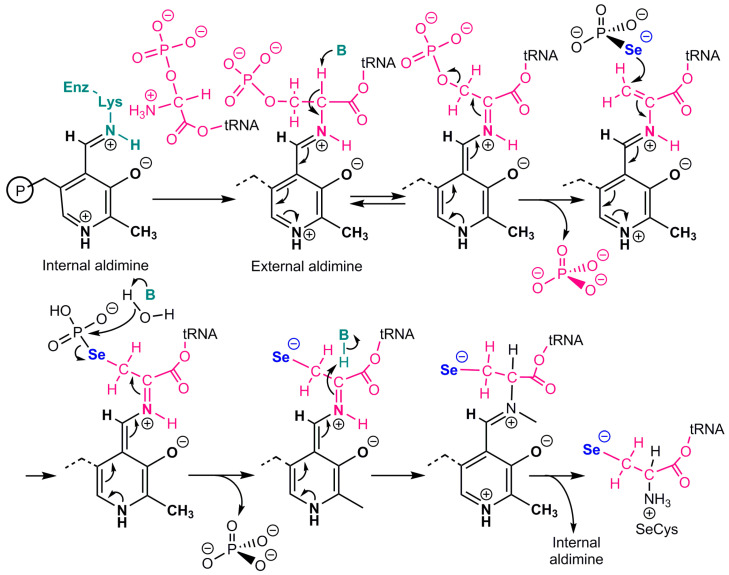
Catalytic mechanism of selenocysteine synthase. Adapted from Ganichkin et al. [26]. tRNA stands for tRNASer/Sec. Pyridoxal phosphate produces an aminoacrylyl-tRNA(Ser)Sec intermediate. Selenophosphate then attacks this electrophilic intermediate, and the resulting selenoester is hydrolyzed to phosphate and selenocysteinyl-t-RNA.

The catalytic mechanism of mouse selenocysteine synthase [26] is shown in Figure 2. This pyridoxal phosphate-dependent enzyme would first produce an aminoacrylyl-tRNA(Ser)Sec intermediate, which agrees with the production of similar intermediates by serine dehydratase [27]. Selenophosphate would then attack this electrophilic intermediate, and the resulting selenoester would be hydrolyzed to phosphate and selenocysteinyl-t-RNA.

Hypermethylation by trimethylguanosine synthase TGS1 of the 5’-cap of mRNAs which code for selenoproteins is linked to the translation efficiency of the UGA/selenocysteine codon in vitro, and it is known that TGS1 activity is required for the biosynthesis of GPx1 in vivo [28]. Additionally, it seems that post-transcriptional modifications of tRNA-(Ser)Sec play a role in the regulation of its transferring activity as a function of selenium availability [24]. 

In mammals, knockout of the gene coding for tRNA(Ser)Sec is lethal. Higher plants, fungi, and many insect species do not have selenoproteins coded in their genome. Human genetic disorders resulting from mutations in genes essential for selenocysteine incorporation have been recently reviewed [29].

Because of distinct contexts of Secys/UGA reading in eucaryotes and procaryotes, the expression of mammalian selenoproteins in *E. coli* has been extremely difficult for many years, but this major difficulty was recently bypassed with a method of recombinant selenoprotein production which uses the amber codon UAG redefined as Sec codon in a specific strain of *E. coli* [30,31,32,33]. Other strategies use engineering of the *E. coli* selenium metabolism along with mutational changes in allo-tRNA and SelA in *E. coli* [34], or genetically encoded photocaged selenocysteines in yeast [35,36,37,38] or in human cell lines [39].

## 3. Mammalian Selenoproteins

Most selenoproteins identified in the living world were eventually discovered thanks to bioinformatic tools developed by Vadim Gladyshev and colleagues [40]. A total of 25 distinct genes coding for selenoproteins have been identified in mammals, and some of them code for several proteins [41]. Knock-out, knock-in, knock-down, and overexpression models plaid a major role in the functional characterization of several of these proteins. When selenium is limiting, the biosynthesis of some selenoproteins is maintained while that of others is not. This results in a well-defined selenoprotein hierarchy in terms of biosynthetic priority and production of selenoprotein m-RNA [42,43], which is generally assumed to reflect the relative biological importance of selenoproteins. When selenium supply is insufficient in cell culture experiments, however, activities of selenoproteins which are low in the hierarchy may be far below their physiological level and lead to misinterpretations. Selenoproteins that rank high in the hierarchy have the most stable m-RNA. Although initially based on radioactive ^75^Se incorporation into selenoproteins, the selenoprotein hierarchy can now be analyzed by monitoring the incorporation of non-radioactive ^76^Se and ^77^Se isotopes using size-exclusion chromatography and mass spectrometry [44].

### 3.1. Selenophosphate Synthetase

Selenophosphate is essential for selenium insertion into biological macromolecules, it is not only used by selenocysteine synthase, but also by enzyme(s] that catalyze sulfur/selenium exchange on thiouridine in tRNA. Selenophosphate synthetase catalyzes the formation of selenophosphate, AMP, and orthophosphate, and it is itself a selenoprotein in some bacteria as well as in archeae and mammals [45,46]. This enigma of chicken and egg is similar to that of the current biosynthetic pathways of coenzymes in which a coenzyme is often required for its own biosynthesis. The enzyme is encoded by selenoprotein D (formerly called selD) gene in prokaryotes, whereas two isoenzymes exist in mammals and in many other animals [47,48]. The first one is a selenium-independent form (SEPHS1), which is also found in animals devoid of selenoproteins and which instead of being involved in selenophosphate synthesis [48,49] would play an important role in redox regulation. The second one is the selenoenzyme SEPHS2 which catalyzes selenophosphate synthesis. In addition to selenocysteine, bacterial SelD, and animal SEPHS2 both contain two essential residues in their active site which are lysine and cysteine.

**Figure 3:**
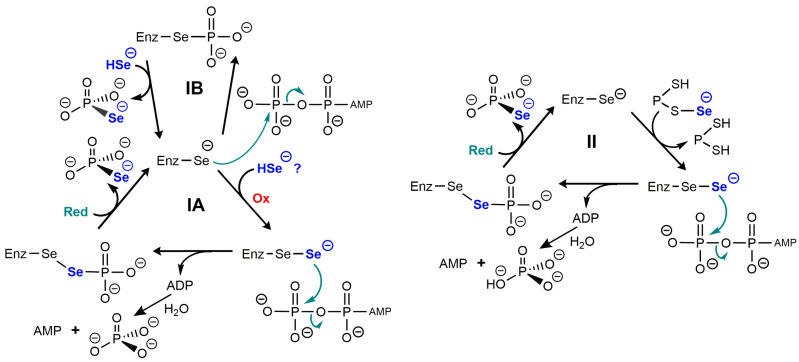
Putative catalytic mechanisms of selenophosphate synthetase. Adapted from studies on the enzyme from *E. coli* [46,50] and that from *Aquifex aeolicus* [45] which bear an essential SeCys residue. In (IA), a perselenide produced by oxidation attacks ATP. The oxidizing cofactor could be O_2_, and the reducing cofactor could be a dithiol such as thioredoxin. (IB) suggests a simpler phosphorylation of SeCys but is not fully supported by structural data [51]. In (II), selenide would be incorporated by a protein partner in the form of a selenopersulfide directly producing a perselenide intermediate [51]. The reducing group “Red” could be a dithiol group of the protein partner.

Some important features of the catalytic mechanism of SelD have been inferred from structural studies, especially those from the enzyme from *E. coli* [46] and from *Aquifex aeolicus* [50]. It was found in vitro that a nucleophilic selenide derivative attacked the gamma phosphorus of ATP to form selenophosphate, while ADP was hydrolyzed to form orthophosphate and AMP [45]. The first mechanistic proposal that was made is summarized in scheme IA of Figure 3.

This mechanism would require an oxidizing cofactor to produce a perselenide and a reducing cofactor, i.e., reduced thioredoxin. With a nucleophilic attack on the γ-phosphorus of ATP, the reason why this mechanism is costing two anhydride bonds of ATP instead of one is not obvious. One speculation is that this would prevent ADP to degrade the selenophosphate bond. One variant of this initial mechanism would involve phosphorylation of the active site selenocysteine (scheme IB) and it seems much simpler, although unfavored by the authors based on structural data [50].

A more important caveat is that the concentrations of HSe^−^ which are required in vitro (based on K_M_ ≃ 7–40 µM) would be highly cytotoxic in vivo, and it has been suggested that it might not be HSe^−^ itself which reacts with the enzyme, but instead a selenium-delivery protein in the form of a selenopersulfide Prot-S-Se^−^ [51]. The protein could be one of the cysteine desulfurases which are known to catalyze the conversion of cysteine into alanine and elemental S. They were shown in vitro to provide inorganic selenium to SelD from selenocysteine. Other ways to produce protein selenopersulfides have been described, for example with rhodanese, from selenite in the presence of GSH [52]. The protein-bound selenopersulfide would then transfer its selenium to the selenocysteine residue of selenophosphate synthase. Such possibilities are illustrated in scheme II of Figure 3.

### 3.2. Selenoprotein P (Selenop)

Selenoprotein P is a mammalian glycoprotein that typically contains 40 to 75% of selenium in circulating blood plasma. It is generally recognized that it serves as a selenium reservoir for the biosynthesis of other selenoproteins [53,54,55,56]. It is the only selenoprotein that contains several selenocysteine residues on a single polypeptide chain, ten residues in human as well as in rat and mouse, and the mode of co-translational insertion of these spaced selenocysteine residues is complex and only partially understood. One knows however that in this case, there are two SECIS elements in the 3’-UTR region, the first one controlling the incorporation of selenocysteine coded by the first UGA, and the next one controlling the incorporation of selenocysteine coded by the other UGAs [24]. Once the first selenocysteine has been inserted, that of the others is much faster [56]. Selenoprotein P is mainly produced in the liver. In extra-hepatic tissues, its capture is under cothe ntrol of lipoprotein receptors. In the kidney, this is an LDL receptor named megalin, which is involved in its capture by proximal tubules [57], whereas in testicles and brain, this is a mechanism of endocytosis that is facilitated by the ApoER2 receptor [58,59]. Cerebral intake of selenium [58] is preceded by the capture of selenoprotein P by cerebral capillaries of the blood-brain barrier. Next, astrocytes that have captured this selenium in a form that remains to be determined synthesize their own selenoprotein P and secrete it in the interstitial medium where it will be used in priority by neurons which are the cells that express most ApoER2.

### 3.3. Selenium-Glutathione Peroxidases (Se-GPx)

Enzymes of the SeGPx family catalyze the reduction of hydroperoxides (H_2_O_2_ and some organic hydroperoxides ROOH) by GSH, and they constitute the largest family of selenoenzymes [60,61]. As shown in Table 1, they include five selenoenzymes in humans [GPx1–4 and GPx6], but it should be underlined that there are three sulfur homologs that have cysteine in place of selenocysteine, GPx5, GPx7 and GPx8, which have much lower or negligible GPx activities but may be involved in antioxidant protection through other enzyme activities.

GPx1, GPx2, and GPx3 are homotetramers, whereas GPx4 is a monomeric enzyme. Cytosolic GPx1 and GPx4 are ubiquitous, whereas GPx2 is mainly found in epithelial cells [62].

**Figure 4:**
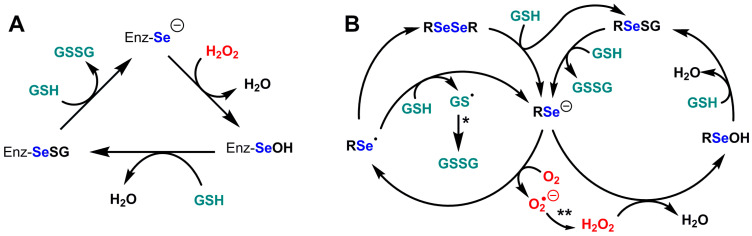
Catalytic cycles of glutathione peroxidases and of the model compound selenocystamine. (**A**) Catalytic cycle of glutathione peroxidases Se-GPx; (**B**) Catalytic cycles of glutathione peroxidase and glutathione oxidase activities of selenocystamine; adapted from Chaudière et al. [192]. (*) GSSG production most likely involves nucleophilic addition of GS^−^ to give a disulfide radical anion GSSG^·−^ which would decompose into GSSG by reducing O_2_ into superoxide O_2_^·−^. (**) The production of H_2_O_2_ from O_2_^·−^ most likely results from dismutation.

Tetrameric Se-GPx has a catalytic cycle that reduces from a kinetic point of view to that of Figure 4A. Everything happens as if no Michaelian complex was formed with each of the two substrates GSH and ROOH (or H_2_O_2_), which means that their rate of decomposition is much faster than their rate of formation. Consequently, such enzymes usually do not exhibit hyperbolic Michaelian rate curves, and they cannot be saturated by their substrates. Their steady-state initial rates are best described by the Dalziel equation [63]:

(E_T_)/V = ϕ_1_/(ROOH) + ϕ_2_/(GSH) + ϕ_12_/[(ROOH).(GSH)] (5)

In physiological conditions for which (GSH) is higher than 1 mM and (ROOH) less than 1 µM, the enzymatic hydroperoxide degradation rate is independent from (GSH).

#### 3.3.1. GPx1

GPx1 is typically the dominant cytoplasmic form in most tissues. In this tetrameric enzyme which exclusively uses GSH in the reduction step of the selenenic intermediate Enz-SeOH, four arginine residues and a lysine residue of an adjacent subunit are involved in the binding of GSH at the active site of each subunit. The enzyme is most efficient to reduce H_2_O_2_ but is also efficient with many organic hydroperoxides [64]. I showed many years ago that mercaptosuccinate was a potent reversible inhibitor of GPx1 [65], and it is often used in cell culture to demonstrate the antioxidant or regulatory role of the enzyme. It was later shown that mercaptosuccinate forms a covalent selenenylsulfide adduct with the active selenocysteine, which eventually results in a sulfenylamide covalent adduct of the active site lysine [66], and it was shown more recently that mercaptosuccinate also inhibits recombinant GPx4 [67].

**Table 1:** Main features of selenium-dependent glutathione peroxidases (Se-GPx) ^1^.

Type	Localization	Structure	Reducing Cosubstrate	Hydroperoxide Substrates	Inhibitors Inactivators	Function ^2^
**GPx1**	Ubiquitous in cytosol and mitochondrial matrix	Homotetramer, five conserved residues at GSH binding site (four Arg, one Lys)	GSH	H_2_O_2_ and alkyl hydroperoxides	Mercaptosuccinate ^3^ O_2_**^.−^**, HOCl Heavy metals	Antioxidant Redox regulator?
**GPx2**	Cytosol of epithelial cells	Homotetramer, extracellular glycoprotein	GSH	H_2_O_2_ and alkyl hydroperoxides	undocumented	Antioxidant Redox regulator?
**GPx3**	Mostly extracell. Blood plasma, Secreted/kidney mammary gland	Homotetramer, Glycoprotein	Thioredoxin; GSH (nonphysiological)	H_2_O_2_ and alkyl hydroperoxides	undocumented	Antioxidant Redox regulator?
**GPx4**	Membrane-bound Cytoplasm Mitochondria Nucleus (minor)	Monomer	Thioredoxin Protein thiols GSH (nonphysiological),	Phospholipid-OOH, Cholesterol-OOH, LDL-OOH	RSL3 ML162	Antiox/Redox regul? Anti-ferroptosis Spermatozoid specific- protein crosslinker
**GpX6**	Olfactory epithelium	Homotetramer, Strong sequence homology with GPx3	Thioredoxin; GSH?	undocumented	undocumented	Antioxidant Redox regulator?

^1^ Essential selenocysteine at the active site. ^2^ The regulatory potential of SeGPx includes down-regulation of NFkB and lipoxygenase activities, and many other controversial targets. ^3^ Recombinant GPx4 would also be inhibited by mercaptosuccinate [67].

GPx1-deficient mice develop normally and show no increased sensitivity to hyperoxia [68], but they are markedly sensitive to H_2_O_2_-induced stress and to neurotoxic compounds such as malonate, 3-nitro-propionate or 1-methyl-4-phenyl-1,2,5,6-tetrahydropyridine [69]. Conversely, overexpression of GPx1 protects mice from acute oxidative stress [70], but these mice later develop hyperglycemia, hyperinsulinemia, increased β-cell mass, insulin resistance, and obesity [71]. This may be since activated oxygen species enhance sensitivity to insulin, as observed in Gpx1^−/−^ mice [71,72,73,74]. The hyperinsulinemic effect of GPx1 overexpression is associated with the upregulation of pancreatic duodenal homeobox-1 (PDX1) and the downregulation of uncoupling protein 2 (UCP2) in pancreatic islets. This at least tells us that persistently overexpressing a selenoenzyme to take advantage of its expected protective effects is potentially dangerous. Interestingly however, β-cell-specific overexpression of GPx1 reverses diabetes in db/db mice [75], which means that systemic and local modulations of GPx1 activity have markedly distinct effects. The explanation is still open to debate [76]. It may involve a major effect of GPx1 overexpression on the downregulation of protein tyrosine phosphatases, including the phosphatase and tensin homolog PTEN, in H_2_O_2_-mediated cell signaling [77,78]. But another rationale comes from a recent and remarkable study which has shown that skeletal muscle NOX4 is responsible for adaptive responses to physical exercise that prevent the development of insulin resistance in mice [79]. This study demonstrates that NOX4-derived H_2_O_2_ is essential to maintain adequate stimulation of Nrf2 with subsequent stimulation of mitochondrial biogenesis and transcriptional activation of antioxidant systems which include SOD2, peroxiredoxins Prdx1 and Prdx6, γ-glutamylcysteine ligase, glucose-6-phosphate dehydrogenase and NQQ1. As well, another important conclusion is that if the activity ratio GPx1/Nox4 is too high, the Nrf2-dependent stimulation of antioxidant protections is compromised, and insulin resistance develops. 

Finally, a decrease in endothelial GPx1 activity may be associated with homocysteinemia which induces arterial alterations and related cardiovascular and cerebral damage. In animal models of homocysteinemia, such as the heterozygous cystathionine-β-synthase-deficient (CBS^+/−^) mouse, a two-fold increase in homocysteine is observed and is associated with impairment of endothelial-dependent relaxation of mesenteric arteries [80] and increased levels of oxidative stress markers such as aortic 3-nitrotyrosine and plasmatic F2-isoprostanes. Similar endothelial dysfunctions were observed in homozygous GPx1-deficient mice [81]. Overexpression of GPx1 in hyper-homocysteinemic mice restored normal endothelial-dependent relaxation and it was shown that the vascular effects of homocysteine were at least partly mediated by impairment of NO production [82,83]. Treatment of cultured endothelial cells with homocysteine did induce a decrease in GPx1 activity [84]. Homocysteine decreases GPx1 expression by a mechanism that does not affect transcription but involves the down-regulation of translation [84].

#### 3.3.2. GPx2

GPx2 is cytosolic and mainly expressed in epithelial cells, especially in the gastro-intestinal system and it uses GSH with high specificity [62,85,86]. Only two of the five residues involved in GSH binding by GPx1 are changed: lysine is replaced by glutamine and arginine is replaced by threonine [60]. GPx2 ranks higher than GPx4 and much higher than GPx1 and GPx3 in the selenium hierarchy of the SeGPx family [60]. A recombinant form of GPx2 has been characterized and found to follow typical ping-pong kinetics with H_2_O_2_-reducing activity being approximately 9-fold lower than those of GPx1 and GPx2 activities [87], but the expression strategy imposed to mutate four cysteine residues into serine. 

Although GPx2 is mainly expressed in the gastrointestinal system, especially by epithelial cells of the esophagus, it is also expressed in other epithelia and in the human liver. It has been proposed that GPx2 prevented the absorption of food hydroperoxides [85], but the highest GPx2 protein concentrations are found in epithelial crypt bases [88] which are not the preferential location for absorption.

GPx2 knock-out mice develop normally [89], but the number of apoptotic cells is increased at crypt bases, even though GPx1 is markedly upregulated in this area [90]. This is an interesting example of partial compensation of GPx2 loss by GPX1 overexpression. Intestinal stem cells are in crypt bases where their growth and differentiation are regulated by the Wnt pathway [91]. Interestingly, GPx2 expression has been shown to be regulated by the Wnt pathway [92,93], which suggests that GPx2 may play a major role in the continuous self-regeneration of the intestinal epithelium.

The redox-sensitive matrix metalloproteinase-7 (MMP-7) which is required to produce microbicidal defensin peptides is modulated by intestine-specific GPx2 [94,95].

GPx2 knock-out mice show an increased allergic airway inflammatory response when challenged with ovalbumin [96], which reflects the fact that GPx2 is also expressed in epithelial cells which do not only belong to the gastrointestinal tract. GPx2 expression is increased in lungs exposed to hyperbaric oxygen [97] or to cigarette smoke [98] and upon treatment of mice with the lung protector and Nrf2 inducer sulforaphane [99].

In mouse embryos, preferential expression of GPx2 was found in rapidly growing tissues, which would again support the idea that GPx2 might play a role in cell proliferation [100]. This physiological function might become deleterious with cancer cells, and GPx2 has indeed been shown to be upregulated in several epithelial cancers [88,101,102,103,104,105,106], which may be all linked to Wnt activation. The link between GPx2-mediated Wnt activation and metastasis and cancer development is supported by recent studies [107,108]. It has also been shown that hydrogen peroxide neutralization by GPx2 is essential for maintaining metastatic capacity in colorectal cancer [109].

GPx2 is also upregulated by the transcription factor ΔNp63 which is abundant in undifferentiated cells [110], and more generally by Nrf2 [111] which stimulates cell protections against electrophilic and oxidative stresses [112]. Just as Wnt activation, Nrf2 activation may have undesirable effects on cancer cell survival [112,113,114,115,116,117], and this dual role of Nrf2 is indeed a reminder of what has been known for years with GSH [118], whose biosynthesis and reductive recycling are stimulated by Nrf2 activation. Finally, GPx2 overexpression is correlated with poor prognosis in patients with hepatocellular carcinoma [119].

The undesirable effect of GPx2 in cancer development may not be trivial, however. For example, GPx activity is known to down-regulate the activities of lipoxygenases [120,121,122], and 12-lipoxygenase promotes epithelial-mesenchymal transition via the Wnt/β-catenin signaling pathway in gastric cancer cells [123].

In addition to its supporting role in cell proliferation, GPx2 has obvious anti-inflammatory properties. Knock-down of GPx2 in adenocarcinoma HT29 cells indeed markedly stimulates the production of COX2 and PGE2 [124]. Conversely, GPx2 is upregulated in models of inflammatory bowel disease and in human colitis [125].

A double knock-out of GPx1 and GPx2 was found to result in chronic colitis [126] and inflammation-driven intestinal cancer [127], mainly caused by Nox1 activation and its associated O_2_^·−^ production [128]. Interestingly, Gpx1^[−/−]^/Gpx2^[+/−]^ mice were unaffected, which means that the GPx2 allele alone was able to prevent intestinal inflammation. Such results demonstrate that weak Gpx2 activity is sufficient to protect against ileocolitis [129].

Another inflammation-linked disease in which GPx2 is involved is viral hepatitis. Oxidative stress is always observed in HCV-infected hepatocytes [130,131,132] and this appears to be largely due to a marked decrease in cellular GPx2 [133]. Such oxidizing conditions are known to stimulate viral protein translation by hepatocytes as well as liver inflammation [134,135]. It has been shown that TP80—which is a retinoid derivative—exhibits an anti-HCV activity which is associated with its restoration of GPx2 activity [136].

#### 3.3.3. GPx3

GPx3 is an extracellular glycoprotein mainly found in blood plasma but also in a few other extra-cellular fluids. It is secreted by the kidney in which the greatest levels of GPx-3 mRNA expression are found, within epithelial cells of the proximal tubule [137,138]. In vitro, GPx3 works well with GSH, but in plasma, GSH concentration is far too low to be used as a reducing coenzyme. GPx3 can use other thiols, for example, one of the reducing proteins thioredoxin, glutaredoxin, or thioredoxin reductase [139] which might be its reducing partners in extracellular compartments. The physiological role of GPx3 is uncertain, however, and this is the selenoprotein that disappears first (bottom of selenium hierarchy) in the situation of selenium deficiency. Some data suggest that an important function of GPx3 might be to preserve nitric oxide bioavailability and to promote an antithrombotic environment [140,141,142]. Other data suggest that GPx3 may behave as a tumor suppressor. In a model of murine inflammatory carcinogenesis, the number of intestinal tumors was indeed doubled in GPx3 knock-out mice [143]. Recent data show however that in a sub-population of murine alveolar epithelial cells from lung tumor, GPx3 has the opposite effect since it promotes metastasis by stabilizing HIF1-α which stimulates the production of Il-10 and thereafter cell migration [144].

#### 3.3.4. GPx4

GPx4, also named PHGPx (Phospholipid Hydroperoxide Glutathione Peroxidase) was initially discovered in pig liver as a peroxidase working on phosphatidylcholine hydroperoxides in liposomes [145]. The sequence of discoveries that followed for four decades was recently summarized by major contributors [146]. This is a monomeric enzyme [147] which together with GPx2 ranks high in the selenium hierarchy of selenoproteins [60] and which occurs in cytosolic, mitochondrial, and nuclear forms. The cytosolic form of the enzyme prevails in somatic tissues, whereas the mitochondrial and nuclear forms are mainly found in testicular tissue [148,149]. The mitochondrial form mGPx4 is identical to the cytosolic form once the mitochondrial targeting sequence has been cleaved. A unique feature of GPx4 is to reduce efficiently lipid hydroperoxides of membranes and lipoproteins, such as hydroperoxides of phospholipids, cholesterol, and cholesterylesters [150,151]. Conversely, H_2_O_2_ and t-butyl hydroperoxide which are good substrates of GPx1 are not efficiently reduced by GPx4. In erythrocytes, hemolysis is inversely related to GPx4 activity during blood bank storage [152].

GPx4 has a unique property which is to accept protein thiols as reducing equivalents when the GSH pool becomes limiting. This situation is observed in spermatozoids during maturation [153,154] and here, oxidation of protein thiols by mitochondrial GPx4 induces the formation of protein polymers through inter-chain disulfides between reduced cysteine residues of GPx4 and cysteine residues of protein partners which have become oxidized to sulfenic acids PSOH. This leads to the fixation of the mitochondrial sheath found in the mid-piece of spermatozoids. Deletion of mGPx4 allows both normal embryogenesis and postnatal development but causes male infertility which is associated with structural abnormalities in spermatozoa [155].

Homozygous Gpx4 knock-in mice obtained by mutation of Sec into Ala are not viable, but studies on heterozygous animals Gpx4^+/−^ have confirmed that GPx4 deficiency is associated with spermatozoid structural abnormalities and reduced motility, and that the resulting infertility of males is markedly counteracted by concomitant knock-out of the Alox15 gene which codes for 15-lipoxygenase [156]. But systemic inactivation of the Alox15 gene does not prevent embryonic lethality in homozygous Sec46/Ala knock-in mice expressing catalytically silent Gpx4 [157]. 

Homozygous GPx4-deficient mice die in utero at midgestation [158,159], and the replacement of native GPx4Se/Se by a cysteine mutant GPx4S/S which conserves a weak but significant GPx4 activity does not prevent the development of embryos, but the animals die from epileptic seizures [160]. GPx4Se/Se is apparently required later for the survival of GABAergic interneurons whose disappearance is lethal.

GPx4 may play a down-regulating role in inflammation. In human dermal fibroblasts which have been transfected to overexpress GPx4, concentrations of phospholipid hydroperoxides are markedly decreased, and when such cells are treated with exogenous phosphatidylcholine hydroperoxides or exposed to UVA irradiation, the activation of the pro-inflammatory transcription factor NFκB and the release of interleukin 6 are also markedly reduced [161].

An active GPx4 is a requirement of a functional CGAS-STING pathway, a vital immune signaling pathway that is activated in response to infection by DNA viruses and which is also involved in tumor surveillance [162,163]. Here the phospholipid hydroperoxidase activity of GPx4 is required to observe the palmitoylation-induced STING activation and its subsequent migration from the endoplasmic reticulum to Golgi.

Finally, one major property of GPx4 is its ability to prevent ferroptosis [160,164]. Ferroptosis is a form of so-called “regulated cell death” which always involves a marked fall in GPx4 activity, and which differs from apoptosis or necrosis in terms of morphological and biochemical features. It mainly relies on the iron-dependent accumulation of lipid peroxides [165,166,167], which would be initiated by two major events, a decrease in GPx4 activity which may or may not be associated with intracellular GSH depletion as well as activation of 12,15-lipoxygenase [168]. Subsequently, non-enzymatic lipid autoxidation would be the final lethal trigger of ferroptosis. Much research work suggests that ferroptosis is involved in many physiopathological situations where GPx4 could be the focus of future drug design [169]. 

Although GPx4 is a monomer, GPx4 allosteric activators have been designed [170]. By increasing GPx4 activity, such allosteric activators suppress ferroptosis induced by erastin and cholesterol hydroperoxides, and they also down-regulate NFkB activation. Additionally, two potent inhibitors of ferroptosis, liproxstatin-1 and ferrostatin-1, have been independently identified in small molecule libraries [171,172]. Liproxstatin-1 is a spiroquinoxalinamine derivative that protects Gpx4 knockout mice from acute renal failure [171]. It was also shown to reduce myocardial infarct size and to restore cardiac GPx4 activity in isolated mouse hearts undergoing post-ischemic reperfusion [173]. The anti-ferroptotic activity of ferrostatin-1 was recently shown to be due to the scavenging of initiating alkoxyl radicals produced by Fe[II] complexes from lipid hydroperoxides [174]. In this process, ferrostatin-1 is not consumed and synergizes with GPx4 to inhibit iron- and hydroperoxide-dependent lipid peroxidation.

A critical discussion of ferroptosis is outside the scope of this review, but one should underline that the concept raises some fundamental questions which are still unsolved [175,176]. Moreover, GPx4 may not be the only selenoprotein whose fall in activity triggers ferroptosis. Given their chloroacetamide warheads, the absolute GPx4 specificity of selenium-targeted inactivators RSL3 or ML162 is unlikely [177,178,179]. Recent experiments show that they do not inhibit purified recombinant GPx4, but instead inhibit thioredoxin reductase 1 [67]. It had been previously shown that the inactivation of GPx4 by RSL3 required the adaptor protein 14-3-3ε in its reduced form [180], which depends on active TrxR1. This suggests that inhibition of TrxR1 might indirectly inhibit GPx4 by preventing the recycling of the reduced form of the adaptor protein [67]. In agreement with the link between ferroptosis and TrxR, another recent publication shows that the natural tetracyclic compound alterperylenol which is an inactivator of TrxR is also an inducer of ferroptosis [181]. Moreover, the idea that inhibition of the glutamate/cystine antiport xCT induces ferroptosis by decreasing GPx4 activity only through GSH depletion is probably an oversimplification. Data show that xCT is required for the extracellular reduction of selenite into selenide, the major form of inorganic selenium that can be internalized by cultured cells [182].

At any rate, GPx4 does prevent ferroptosis, and this may inspire cytoprotective pharmacological strategies, but this may also have deleterious effects on tumor growth and anti-cancer therapy. This is most obvious in epithelial-derived carcinomas in which tumor cells have been fixed in a therapy-resistant mesenchymal state which clearly relies on GPx4 expression [183,184]. In such extensive studies, statins—which inhibit GPx4 biosynthesis—were shown to decrease tumor cell resistance to cytotoxic antitumor drugs by synergizing with GPx4 inhibitors such as RSL3 [185]. These in vitro observations were also fully supported by in vivo experiments on tumor xenografts built from GPx4-wild type and GPx4 knock-out clones of such cells. The EMT regulator ZEB1 was strongly correlated with mesenchymal state sensitivity to GPx4 inhibition, and ZEB1 deletion abolished sensitivity to GPx4 inhibition. ZEB1 regulates the uptake and mobilization of lipids and affects the EMT-associated remodeling of sphingolipids in the plasma membrane [186], a process that plays a central role in intercellular connections.

Clear-cell carcinomas (CCC) are therapy-resistant malignant cells observed in a variety of cancers, most frequently in kidney and ovarian cancers. It was found that inhibitors of GPx4 were potent and selective killers of such cells [187], and this was related to their HIF2α-dependent enrichment in polyunsaturated lipids which made them highly vulnerable to ferroptosis.

#### 3.3.5. Other Members of the GPx Family

GPx6 is the last member of the SeGPx family in humans, but it is not a selenoprotein in rats and mice where selenocysteine has been replaced by cysteine [60]. Human GPx6 exhibits a strong sequence homology with plasmatic GPx3, and it is expressed in the olfactory epithelium.

Finally, many homologous sequences of Se-GPx in which selenocysteine is replaced by cysteine have been identified in in non-vertebrate animals, higher plants, and fungi. Such sulfur homologs of Se-GPx are not all involved in hydroperoxide reduction, and most of them do not use GSH as a reducing co-enzyme but use instead the active-site cysteine dithiol group of thioredoxin, other redoxins, or protein disulfide isomerase [60,188,189,190], as further discussed in Section 6.

In mammals, GPx7 belongs to the CysGPx family, but it has a strong sequence homology with GPx4, and it also adopts a monomeric structure. It is localized in the lumen of the endoplasmic reticulum of epithelial cells where it acts as a catalyst of protein refolding, in association with protein disulfide isomerase [190]. In this mechanism, the active site cysteine thiol of GPx7 would be oxidized by hydroperoxide into a sulfenic acid intermediate PSOH which in turn would oxidize protein disulfide isomerase. But GSH and protein disulfide isomerase would compete as reducing cofactors of GPx7 in the endoplasmic reticulum [191].

#### 3.3.6. Mechanistic Complexity of Glutathione Peroxidases

**Figure 5:**
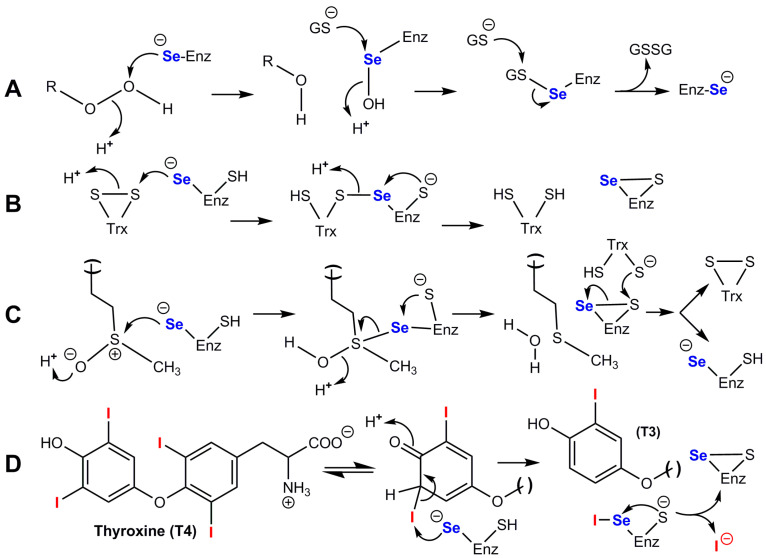
Catalytic mechanisms of the four main families of selenoenzymes in mammals. (**A**) Glutathione peroxidases SeGPx. (**B**) Thioredoxin reductases TrxR; the selenenylsulfide group present in the C-terminal region of a subunit is reduced to a selenol/thiol group by a dithiol group in the N-terminal region of the other subunit; the resulting disulfide is then recycled by flavin/NADPH. (**C**) R-methionine sulfoxide reductase MsrB1; a selenenic acid intermediate cannot be excluded in the second step. (**D**) Deiodinases DIO1 and DIO2; the reduced enzyme is recycled by thioredoxin or by glutaredoxin/GSH.

The generic mechanism of Se-GPx (see Figure 4A) does not exclude some surprises. For example, is the reactivity of the resting selenium with oxygen always negligible? with an apparent pKa of SeCys which is presumably lower than 5.5, it is usually postulated that the basic and highly reducing selenolate form is dominant in the resting enzyme. Selenolates can catalytically reduce dioxygen O_2_ into superoxyde O_2_^·−^ in the presence of GSH, which we first showed with selenocystamine [192] and which turned out to be a common feature of alkyl- and aryl-selenolates. Following the discovery of the GPx activity of ebselen [193,194], many biomimetic selenium catalysts have been studied, but only two of them—ebselen and BXT-51072—have been tested in clinical development, because of their anti-inflammatory properties and their very low toxicity [195,196,197]. In both cases, we observed that the peroxidase/oxidase ratio was high. Our study of half a dozen model compounds led to the conclusion that the rate of oxygen reduction in the presence of GSH was a major factor of cytotoxicity [197,198]. One cannot exclude the possibility that some GPx would maintain the selenocysteine residue in its protonated selenol form to avoid oxygen one-electron reduction in the resting enzyme. Additionally, the production of a selenenic enzyme intermediate EnzSe-OH in the hydroperoxide-reduction step (Figure 4A and Figure 5A) seems unavoidable, but the reactivity of selenenic acids is so high that nobody ever observed the presumed selenenic intermediate in SeGPx active sites by mass spectrometry or spectrophotometric techniques. Sulfenic acid traps such as cyanide or dimedone have no effect on the enzyme but may be inappropriate for selenenic trapping [199,200]. At least one successful identification of a selenenic acid intermediate was obtained with dimedone for selenoprotein S, but this required that fast selenenic acid trapping as an internal selenenylsulfide be prevented by cysteine-to-serine mutation [186]. Direct observation of a transient selenenic acid has however been made in an ingenious molecular model [201].

### 3.4. Thioredoxin Reductases (TrxR)

TrxR are NADPH-dependent enzymes that catalyze the reduction of the internal disulfide of thioredoxins (Trx) into a biologically active dithiol [202,203,204]. Trx are themselves 12-kDa reductases that catalyze the conversion of disulfide to dithiol in other protein targets and which have a conserved -Cys-Gly-Pro-Cys- active site motif [205,206,207]. They are ubiquitous in the living world, from archaea to mammals. Trx was first purified and characterized as the hydrogen donor for ribonucleotide reductase [RNR] in *Escherichia coli* [208,209]. This observation was later extended to mammals, confirming the importance of this reducing protein in DNA synthesis. In mammalian cells, there are two isoforms of Trx, Trx1 which is mainly cytosolic but can be translocated into the nucleus and sometimes secreted out of the cell, and mitochondrial Trx2.

Mammalian TrxR has a selenocysteine essential residue at the active site, which is not the case in prokaryotes. They include three selenoenzymes in humans, the cytosolic TrxR1, the mitochondrial TrxR2, and a specific isoenzyme TrxR3 also known as TGR in testicles. Each of the three enzymes includes isoforms due to extensive splicing. 

Many questions are still puzzling concerning the relative importance of the three TrxR families. For example, knock-out models have shown that TrxR1 is essential for brain development, which is surprisingly not the case for TrxR2 [47].

As a rule, they play a major role in redox regulation of metabolism and cell functions, because Trx is used as a reducing cofactor of enzymes such as ribonucleotide reductase, peroxiredoxins, methionine sulfoxide reductases, and GPx3, and it interacts with many other target enzymes in which cysteine residues have been oxidized [210]. Such cysteine residues are normally reduced in their resting form but may be oxidized into sulfenic acid PSOH or disulfide during redox signaling or because of oxidative stress.

Although TrxRs are considered the major reducing system for thioredoxin, thioredoxin may also be reduced by the glutaredoxin/GSH couple which would act as a backup system [211,212]. This could only result in partial compensation, however, because the inactivation of each of these two systems leads to many distinct effects and to a large extent opposite metabolic effects [213]. A good illustration of this is that Trx is maintained in its reduced form in mouse embryonic fibroblasts lacking TrxR1, but that this is not the case if the cells are incubated with high glucose concentrations [214].

TrxR is usually antiparallel homodimers [215,216]. They have much structural homology with other NADPH- and flavin-dependent disulfide reductases such as glutathione reductase. The main difference is due to the presence of a C-terminal extension containing a Gly-Cys-Sec-Gly motif [215]. The catalytic mechanism of mammalian TrxR [202,216,217,218,219] involves the transfer of electrons from the active site flavin FADH_2_ to an internal disulfide of the N-terminal domain of the same subunit. The resulting dithiol motif -Cys-Val-Asn-Val-Gly-Cys—is not interacting with the target protein used as a substrate, it rather transfers its electrons to the selenenylsulfide group present in the C-terminal region of the other subunit. This finally produces a selenol/thiol entity which is then responsible for the reduction of most substrates of the enzyme(s), which include Trx but also artificial disulfide such as 5,5’dithiobis[2-nitrobenzoate] known as DTNB. NADPH is of course used to recycle FADH_2_. In this mechanism, a cysteine selenolate performs the final reduction of the disulfide substrate as illustrated in Figure 5B. Mutation of selenocysteine into cysteine results in an enzyme whose kcat is 100-fold lower than that of the wild-type selenoenzyme [220].

A distinctive mechanistic property of TrxR, compared with other selenoenzymes, is their ability to catalyze one-electron reductions. It was shown that TrxR2 was able to reduce ferric cytochrome c, ascorbyl radical, and dehydroascorbate by means of one-electron transfers [221]. Cysteine mutants did not conserve these properties, which means that in addition to reduced flavin which is known to be a one-electron-reducing coenzyme, selenium is essential for subsequent one-electron transfers. One exception is known however with juglone [5-hydroxy-1,4-naphthoquinone], a walnut toxin that is reduced by TrxR1 in a one-electron transfer which does not require selenocysteine and is coupled to the reduction of oxygen to superoxide [222].

TrxR is not fully dedicated to Trx reduction [223]. For example, TrxR1 also catalyzes the reduction of protein disulfide isomerase PDI, glutaredoxin Grx2, and small natural molecules such as selenite and lipoic acid [224]. Similar with selenoprotein P, we are dealing here with a selenoenzyme which may play a role in its own production since it is involved in the formation of hydrogen selenide from selenite. The easy accessibility of the highly reactive selenocysteine of TrxRs should explain their wide substrate specificity [223].

By ensuring the reduction of thioredoxins, TrxR is unavoidably major actors of a cascade of redox regulations in mammalian cells, with many protein targets of thioredoxins having essential functions. These redox-sensitive proteins form redox-dependent signaling pathways that are crucial for fundamental cellular processes, including metabolism, proliferation, differentiation, migration, and apoptosis [205].

As already mentioned, thioredoxin may be the main reducing cofactor of GPx3, but the biological link between TrxR1 and Gpx3 is not well documented. Thioredoxin is also the reducing cofactor of peroxiredoxins [205] and methionine sulfoxide reductases [225,226,227].

Peroxiredoxins have a cysteine-containing active site that catalyzes the intracellular reduction of H_2_O_2_ for signaling purposes [228] and act as extremely efficient H_2_O_2_ sensors. The active sites of Prx have an affinity for H_2_O_2_ which seems to have no equivalent in the world of peroxidases or a fortiori in that of catalases, and this markedly accelerates the oxidation of their essential cysteine. Second-order rate constants for the reduction of H_2_O_2_ by peroxiredoxins are close to 10^7^ M^−1^.s^−1^, which is more than three orders of magnitude higher than those of small molecular-weight mercaptans. The role of Prx in H_2_O_2_-dependent signal transduction relies on the oxidation of target proteins. We do not know however if this is a generic function of Prx or only a specialized function of some of them.

It is not excluded that Prx also works as H_2_O_2_-dedicated antioxidants since they are abundant in mammalian cells. They are also able to reduce some hydroperoxides other than H_2_O_2_, although the physiological significance of these weaker activities is unknown. The distinctive feature of peroxiredoxins compared with other hydroperoxide reductases is that their active site does not require selenocysteine but only a dithiol that reduces their substrate with concomitant formation of an intersubunit disulfide [205]. Because TrxR reduces Trx which reduces Prx, they are unavoidably involved in the redox regulation of Prx, but other levels of Prx regulation involve kinase-mediated tyrosine phosphorylation [229] and H_2_O_2_-mediated hyperoxidation. Hyperoxidation of Prx occurs slowly when continuous exposure to H_2_O_2_ oxidizes the N-terminal catalytic cysteine into a sulfinic acid [230]. Trx is not able to reduce this sulfinylated form back to thiol, but this is achieved by sulfiredoxin Srx [231]. Hyperoxidation of Prxs leads to loss of peroxidase activity and stimulates chaperone activity [232]. Sulfinylated Prx activates transcription factors involved in sulfiredoxin biosynthesis [229].

Methionine sulfoxide reductases [225,227,233,234] are also using thioredoxin as a reducing cofactor, and they include the selenoenzyme MsrB1 discussed in the next section.

TrxR1 suppresses adipocyte differentiation and insulin responsiveness [235], which suggests that TrxR1 has synergistic effects on the antioxidant function of GPx1. The fact that TrxR1 protects tyrosine phosphatase 1B from inactivation by H_2_O_2_ also supports this hypothesis [236].

“Redox regulation” is probably the best functional descriptor of enzymes of the TrxR family, just as “antioxidant hydroperoxide degradation” is the best descriptor of enzymes of the SeGPx family. But this does not mean that SeGPx do not have a role in redox regulation, and here with TrxR, an important question is that of their role in antioxidant protection. The answer is that thioredoxin and thioredoxin reductases do have antioxidant functions [202], but those which are consequences of their regulatory functions may be as important as those which come from their intrinsic antioxidant properties as reducing cofactors of protective enzymes. Nrf2 is one of the regulatory targets which supports this concept. The idea that TrxR1 is probably a major gatekeeper of Nrf2 activation has been developed in a comprehensive review of TrxR1-Nrf2 interactions [204]. Inhibition of TrxR1 markedly activates Nrf2 which is the major transcriptional regulator of cellular responses to oxidative and electrophilic stress. As well, reciprocally, Nrf2 activation by electrophilic or oxidative stress induces the expression of both Trx and TrxR1.

TrxR1 is, however, itself a sensitive target of electrophilic species, and some of them, such as cis-diaminedichloroplatinum, transform the enzyme into SecTRAPs (selenium-compromised thioredoxin reductase-derived apoptotic proteins) which behave as harmful NADPH oxidases [237,238]. This is because they have lost their selenocysteine-dependent reducing properties but have kept their other redox components which are now able to catalyze redox cycling of quinonic substrates.

**Figure 6:**
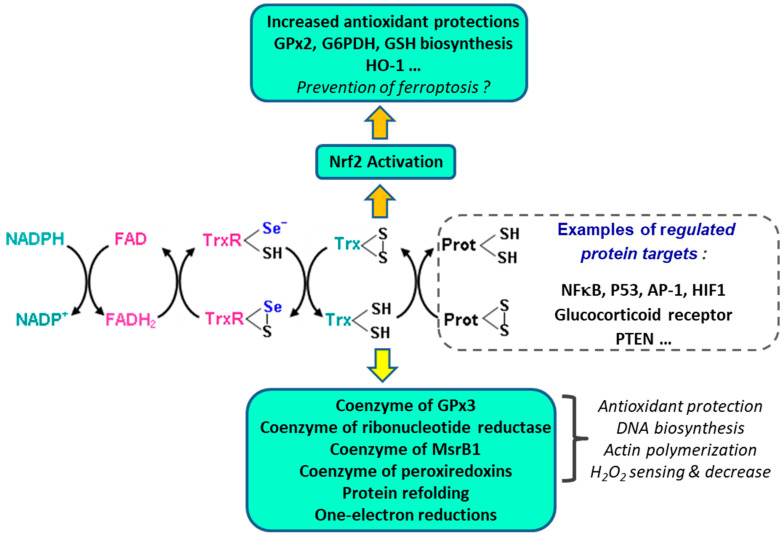
The TrxR/Trx system and its redox network of protein regulations. The reduced and oxidized forms of thioredoxin are involved in antioxidant protection, cell division, control of cytoskeleton structure, apoptosis, inflammation, and redox control of many regulatory proteins.

The complex redox network in which the TrxR/Trx system is involved is summarized in Figure 6. Trx1 can be translocated to the nucleus where it regulates the activity of several transcription factors which include Nrf2 [204,239], NFkB [240], p53 [241], AP-1 [242], and HIF-1 [243], as well as that of the glucocorticoid receptor [244]. The reduced form of Trx1 inhibits the phosphatase activities of the tumor suppressor PTEN, thereby stimulating cell proliferation and tumor growth [245]. PTEN (Phosphatase and Tensin Homolog 1) is a multi-domain protein that exerts its tumor-suppressive functions in a lipid phosphatase-dependent, protein phosphatase-dependent, or scaffold-dependent manner [246].

A final comment is required on the role of TrxR in cancer. In many tumors, TrxR1 is overexpressed, and they are believed to stimulate proliferation and inhibit apoptosis in cancer cells. This is why they have been considered an interesting target in cancer therapy [247]. An efficient and tolerable therapeutic window of TrxR inhibition may however be difficult to establish. It has been reported that 90% knocking down of TrxR1 has a negligible effect on cell growth in a human carcinoma cell line expressing high levels of TrxR1 [248], and it was also reported that 90% pharmacological inhibition of Trx1 had no effect on oxidized Trx1 in HeLa cells [249]. Such observations suggest that residual amounts of TrxR1 may be sufficient to maintain its essential functions in tumor cells. Nevertheless, intensive activities of anti-tumor drug design are currently going on in this area [250,251].

Trx1 is also regulating the apoptosis signal kinase 1 (ASK1). In its reduced form, Trx1 inhibits ASK1 and prevents apoptosis, whereas in the situation of oxidative stress, its oxidized form dissociates from ASK1 which results in apoptosis [252]. ASK1-Trx1 dissociation is not only achieved by oxidation but can also be induced by the Trx1-interacting protein TXNIP which competes with ASK1 by binding to the reduced form of Trx1 [253].

### 3.5. Selenium-Dependent Deiodinases

Selenium-dependent deiodinases catalyze the reductive deiodination of thyroxine [T4] or triiodothyronine [T3]. In mammals and in humans, they include three selenoenzymes [254,255,256,257,258], which are homodimeric structural homologs of thioredoxins [257,258,259]. They rank high in the selenium dependence hierarchy, such as GPx2 and GPx4.

**Figure 7:**
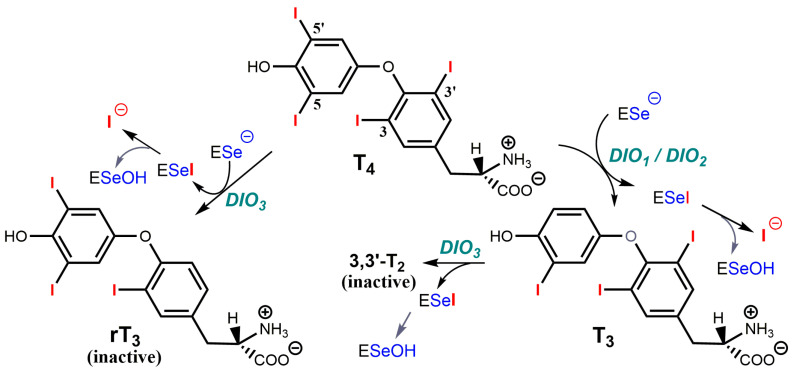
Reactions catalyzed by the three selenium-dependent deiodinases. DiO1 and DiO2 produce the active hormone T3 from T4, whereas DiO3 produces two inactive metabolites, rT3 [reverse T3] from T4, and 3,3′-T2 from T3.

The thyroid gland secretes a pro-hormone, tetra-iodothyronine, or thyroxine T4 which in target cells of peripheral tissues is converted into triiodothyronine T3, the active hormone. As shown in Figure 7, type 1 and type 2 deiodinases [DIO1 and DIO2] both produce T3 from T4, whereas type 3 deiodinase (DIO3) converts T4 into the inactive metabolite rT3 [reverse T3], and T3 into inactive 3,3′-T2. Selenium-dependent deiodinases, therefore, control the activity of T3, which interacts with nuclear receptors TRα and TRβ, and which is a major effector of lipid and sugar metabolism, as well as of respiration and mitochondrial biogenesis [260]. For example, the induction of DIO2 in brown adipose tissue exposed to cold stimulates energetic expenditure. This DIO2 increase is under the control of cAMP which binds regulatory subunits of PKA [259] and releases activated catalytic subunits. Conversely, DIO3 in the ischemic/hypoxic heart or brain decreases the energetic expenditure to cope with decreased oxygen delivery, and this other process is under the control of HIF-1α which interacts with the DIO3 promoter [259].

DIO1 is a thyroxine 5’-deiodinase that is associated with the plasma membrane in cells of the liver, kidney, thyroid, and pituitary gland, whereas DIO2 is a thyroxine 5’-deiodinase that is associated with the endoplasmic reticulum and cells of the pituitary gland, thyroid, skeletal muscles, brown adipose tissue, heart, and CNS.

DIO2 expression is tightly regulated by transcriptional mechanisms [258] and deactivated by ubiquitination [261].

The three iodothyronine deiodinases catalyze similar reactions with distinct regiospecificity. Their structural and mechanistic analysis as integral membrane proteins has been difficult [255]. The crystal structure of the truncated catalytic domain of mouse Dio3 has been solved [256], and it shows that the enzyme has a close structural similarity to atypical 2-Cys peroxiredoxins. The structure is compatible with Sec170 extracting the 5-iodine as a selenenyl-iodide, which should then hydrolyze to a selenenic acid intermediate, as supported by model studies with a synthetic alkylselenenyl-iodide [262]. It also shows that the thyronine ring is protonated via a network of conserved amino acids. The oxidized enzyme can be directly reduced by exogenous dithiols in vitro, and it is reduced by physiological concentrations of either thioredoxin or glutaredoxin. There are still unanswered questions about possibly distinct catalytic and regulatory mechanisms of the three deiodinases [263]. In the catalytic mechanism of T4 to T3 conversion, which is illustrated in Figure 5D, an enzyme-catalyzed keto-enol tautomerization would fit with a direct attack of iodine by selenium with regiospecific proton exchange at the active site of DIO1 or DIO2. In the conversion of T4 to rT3 by DIO3, such a tautomerization mechanism seems unlikely because it would involve a much more unstable oxonium intermediate, but one cannot exclude its stabilization through electrostatic interactions. In all cases, the internal selenenylsulfide which is produced is not reduced by glutathione GSH. It may be first reduced by a second proximal cysteine residue to form a mixed disulfide, but the reductant which can recycle the active site in vitro must be a dithiol such as a dithiothreitol or dithioerythritol which does not have the constraints of charges and steric hindrance of GSH. The physiological reduction is unknown but may be performed by thiol groups of proteins such as the Trx/TrxR system or the glutaredoxin/GSH system [256,257]. 

To complete our overview of selenoproteins in thyroid iodinated hormone production, we should underline that thyronine iodination to T4 is achieved on the thyroglobulin protein within the thyroid gland by means of thyroid peroxidase (TPO) which uses iodide I^−^ and H_2_O_2_ as cosubstrates [264,265]. Thyroxine T4 is then cleaved by proteolysis of thyroglobulin [266] within the thyroid gland, and then secreted into blood plasma and transported to peripheral tissues by specific carriers. It is then taken up by an anion transporter of its target cells where it diffuses to reach its nuclear receptor. The secretion of T4 is under the control of the hormone TSH which is produced by hypophysis.

H_2_O_2_ which is used by TPO is generated by NADPH oxidases DUOX1 and DUOX2, and its local concentration in the thyroid gland might be under the control of GPx1, GPx3, and TrxR, GPx4 being additionally present. Thus, the biosynthesis of T4 and its conversion into T3 might be under the control of half a dozen selenoproteins.

Congenital hypothyroidism is the most common congenital endocrine disorder in humans, and some of the associated genetic defects which have been identified include recessive mutations in the selenocysteine insertion sequence SBP2 [267], but genetic defects of the selenium-dependent deiodinases are not clearly documented. The main genetic defects associated with hypothyroidism bear on mutations of thyroglobulin, TPO, DUOX2, and tyrosine deiodinase [267,268] which are not selenoenzymes. The most characteristic thyroid phenotype exhibits low serum T3, high T4, and high rT3.

### 3.6. Methionine R-Sulfoxide Reductase B1 [MsrB1]

Methionine sulfoxide reductases catalyze the reduction of sulfoxide into thioether, using thioredoxin Trx as a reducing cofactor [269,270,271,272]. Methionine sulfoxide has a chiral center which is sulfur, and MsrA is preferentially reducing the S form whereas MsrB is preferentially reducing the R form. In addition to MsrB1, selenocysteine-containing MsrA has been identified in bacteria, algae, and invertebrate animals, but not in vertebrates [271]. They are extremely rare and not well understood. Most MsrA have an essential cysteine at the active site.

Among MsrB which have been identified in animals and especially in humans, only MsrB1 (formerly named selenoprotein R or selenoprotein X), is a selenoenzyme [219,269,270,271,272], and it is also the only one that has a strict specificity for the R form. 

Sulfur homologs of MsrB1 exist, in which selenocysteine is replaced by cysteine, but their specific activities are 100- to 1000-fold smaller. Conversely, replacing the essential cysteine of MsrB2 and MsrB3 with selenocysteine increases their specific activities by more than 100-fold. Such observations confirm the catalytic advantage of selenium. Human MsrB1 has two Cys-X-X-Cys motifs that coordinate a zinc atom, but the latter is found in other MsrB and it is apparently not involved in catalysis. Upon reduction of methionine sulfoxide, a selenenylsulfide bond is formed and then reduced by thioredoxin [226,270,271]. Although not excluded, a selenenic acid intermediate has not been demonstrated. The main features of the catalytic mechanism of MsrB1 are summarized in Figure 5C.

Despite its former recognition as an antioxidant enzyme in MsrB1^−/−^ mice [273], MsrB1 was found to perform a stereospecific reduction of each of the two methionine-R-sulfoxide residues which are produced on actin filaments by flavin-dependent monooxygenases of the MICAL family [234,274]. This MICAL-dependent oxidation triggers the dissociation of polymeric F-actin into globular monomers of G-actin, and conversely, MsrB1 triggers the polymerization of G-actin into F-actin. Thus, the MICAL/MsrB1 couple plays a central role in the regulation of the polymerization/depolymerization process, and it is involved in many physiological events such as cell division, cytokinesis and cell contractions, as well as in hair development or muscle organization [272].

The MICAL/MsrB1 couple is likely to play important roles in physiopathology. For example, a strong increase in MsrB1 protein and MsrB activity is observed in LPS-treated macrophages where MsrB1 colocalizes with actin [275]. As well, MsrB1 has “anti-inflammatory properties” since LPS-treated MsrB1^−/−^ macrophages have greatly reduced anti-inflammatory cytokines, such as Il10 and Il1rn, but increased pro-inflammatory cytokines such as Il12A and Il12B [276]. In the heart, the MICAL/MsrB1 couple regulates the Ca^2+^ calmodulin-dependent kinase (CAMKII). MICAL oxidizes methionine M308 of CAMKII, which prevents calmodulin binding and kinase activity [277].

Surprisingly, however, MsrB1^−/−^ mice do not show strong phenotypic alterations [273], which suggests that compensatory activities may exist. Moreover, a quadrupole KO for MsrA/MsrB1/MsrB2/MsrB3 paradoxically results in mice more resistant to cardiac ischemia/reperfusion or treatment with paraquat [278], which is not explained, although suggesting some link with superoxide production or toxicity.

### 3.7. Other Mammalian Selenoproteins

Although there are still a few mammalian selenoproteins for which little information is available, our understanding of several mammalian selenoproteins substantially improved in recent years.

#### 3.7.1. Selenoprotein O and Protein AMPylation

Selenoprotein O is a large size selenoprotein (73 kDa for the human mitochondrial protein) that is highly conserved in bacteria and eukaryotes. It belongs to the superfamily of pseudokinases which are generally believed to have non-catalytic functions, such as allosteric regulation or scaffolding [279]. It was found however that selenoprotein O is an active enzyme that transfers AMP instead of phosphate, to serine, threonine, or tyrosine residues of protein substrates [280]. Structural data obtained with *P. syringae* selenoprotein O show that it adopts a protein kinase-like fold in which ATP is positioned in a head-to-tail configuration, which explains why this is the α-phosphate (and its adenylyl substituent) which is transferred to protein targets [280]. Here, the γ-phosphate which is normally transferred by protein kinases is buried in an inaccessible pocket, which is largely due to its coordination by a highly conserved lysine residue. It was also found with *E. coli* selenoprotein O that the AMPylation activity required the reduction of an intramolecular disulfide bridge. In this work, selenoprotein O was further shown to protect S. cerevisiae in situations of oxidative stress triggered by either H_2_O_2_ or menadione. One of the protein substrates which was shown to be AMPylated by selenoprotein O is glutaredoxin which is known to remove glutathione from S-glutathionylated proteins. Reversible S-glutathionylation is an important mechanism of transient protection of proteins from overoxidation [281]. Under exposure of *E. coli* or yeast cells to GSSG or diamide, it was shown that the overall level of S-glutathionylation was markedly decreased in selenoprotein-O KO cells [280]. Thus, selenoprotein O-mediated AMPylation of glutaredoxin family members would regulate the S-glutathionylation of proteins in vivo.

#### 3.7.2. Selenoprotein F and Endoplasmic Reticulum Glycoproteins

Selenoprotein F (previously called Sep15) is a 15 kDa protein which is an endoplasmic reticulum-resident protein, and which forms a 1:1 complex with UGGT (UDP-glucose:glycoprotein glucosyl transferase) [282]. It has a thioredoxin fold with selenocysteine present in the position of the redox-active cysteine of thioredoxin and a reduction potential of −225 mV/ENH, between that of thioredoxin and that of protein disulfide isomerase, which strongly suggests a redox function [283]. Data obtained in mice have shown that selenoprotein F plays the role of “gatekeeper” of disulfide-rich glycoproteins secreted by the endoplasmic reticulum [284]. This function would prevent the useless secretion and costly resynthesis of glycoproteins in which disulfide formation is incorrect, such problems being well documented in the ER. The underlying mechanism is not well understood, however.

It was later shown that selenoprotein-F knockout mice developed nuclear cataracts at an early age, and it was assumed that this might be due to improper folding of lens proteins [285]. In a more recent study, it was shown that such selenoprotein-F knockout mice developed glucose intolerance and insulin reduction and that the deleterious effects induced by a high-fat diet (obesity, hyperglycemia, glucose intolerance, and hepatic steatosis) were markedly increased [286].

#### 3.7.3. Selenoprotein N and Endoplasmic Reticulum Calcium Sensing

Selenoprotein N (also called SEPN1) is a 70 KDa transmembrane glycoprotein of the endoplasmic reticulum which contains one selenocysteine residue [287]. Mutations of the SELENON gene lead to myopathy of variable severity. High levels of selenoprotein N are observed in human fetal tissues and its high expression in cultured myoblasts is downregulated in differentiating myotubes, which suggests a role in cell proliferation. The sarcoplasmic/endoplasmic reticulum Ca^2+^-ATPase (SERCA) mediates calcium uptake in the endoplasmic reticulum, whereas calcium release is mediated by inositol triphosphate and ryanodine receptors. The overall process must be tightly controlled, to maintain the steep calcium gradient required across ER and thereby rapid excitation/contraction coupling. It has been known for a long time that this is a redox control. The latter involves the dissociation of a disulfide of SERCA2, which in the oxidative conditions which prevail in the lumen of the ER, would require a reductase that was not identified [288]. A recent study shows that the N-terminal domain of selenoprotein N senses ER calcium fluctuations and that low luminal calcium triggers the dissociation of an oligomeric form of SEPN1 into monomers, which concomitant unmasking of its reductase activity toward protein CERCA2 [289]. Thus, selenoprotein N would be one of the long-sought reductases which mediate the replenishment of ER calcium stores during muscle contraction.

#### 3.7.4. Selenoprotein I and Ethanolamine Phospholipids

Selenoprotein I is an ethanolamine phosphotransferase (EPT) involved in the synthesis of two distinct ethanolamine phospholipids, phosphatidylethanolamine (PE) and plasmenyl PE [290]. These transferase activities are not redox reactions and do not use the selenocysteine residue. The selenocysteine which is in the C-terminal region serves an unknown function. Deletion of SELENOI in mice prevents embryo development, while loss-of-function mutations of SELENOI in humans lead to hereditary spastic paraplegia [291]. The enzyme is required in the myelination process and neurodevelopment, and in the maintenance of normal homeostasis of ether-linked phospholipids in humans [291,292]. Selenoprotein I also plays an important role in metabolic reprogramming required for T cell activation/proliferation and optimal immunity [293]: T cell activation increases levels of selenoprotein I along with PE and plasmenyl PE, and selenoprotein-I deficiency in mouse T cells reduces the de novo synthesis of PE and plasmenyl PE as well as proliferation. T-cell activation in selenoprotein-I knockout resulted in an accumulation of ATP and decreased AMPK activation, which disrupted metabolic reprogramming and reduced cell cycle progression.

#### 3.7.5. Selenoprotein K, Palmitoylation Cofactor and Inhibitor of ER-Induced Apoptosis

Selenoprotein K is a 10 KDa transmembrane protein associated with the endoplasmic reticulum, with a selenocysteine in the C-terminal region. It serves as a cofactor during protein palmitoylation by binding to the protein acyltransferase DHHC6, facilitating the addition of palmitate via a thioester bond to the sulfhydryl group of cysteine residues of target proteins [294]. This is illustrated by the essential role of a selenoprotein-K/DHHC complex in the palmitoylation of IP3 receptors in immune cells [295]. Thus, through specific interactions with distinct proteins, some selenoproteins may serve as cofactors of enzyme reactions rather than being used as selenoenzymes.

SELENOK gene knockout was shown to increase endoplasmic reticulum stress and induce apoptosis in neurons in vitro and in vivo, through intracellular calcium increase and activation of the m-calpain/caspase-12 cascade [296]. It was also shown that selenoprotein K expression was activated in myogenic cells during differentiation in vitro and in vivo [297]. Here, SiRNA-mediated silencing of selenoprotein K inhibited the development of myoblasts into myotubes and concomitantly increased apoptosis and autophagy in myogenic cells.

#### 3.7.6. Selenoprotein W and EGF Regulation

Selenoprotein W is a cytoplasmic 9kDa thioredoxin-like protein that is required for cell cycle progression [298,299,300], and it has been shown that it is required for EGF-induced EGFR activation through suppression of EGFR ubiquitination and receptor degradation [301]. Interestingly, it interacts with the adaptor protein 14-3-3b [302]. This interaction requires the selenocysteine residue and is increased by diamide, which suggests that selenoprotein W is redox regulated.

#### 3.7.7. Selenoprotein T

Selenoprotein T is a thioredoxin-like protein that is mostly expressed in endocrine organs, and which is involved in the production and release of hormones such as insulin and corticotropin [303]. Such effects are mediated by its role in ER proteostasis. It is essential during embryogenesis, and its knock-out in the mouse brain induces anatomical alterations and abnormal behavior. Recent experimental data support the involvement of selenoprotein-T dysfunction in Parkinson’s disease [304,305].

#### 3.7.8. Selenoprotein S

Selenoprotein-S is a protein of 189 amino acids which is prevalent in eukaryotic organisms. It is an intrinsically disordered membrane enzyme whose function is not established, but which is associated with the unfolded protein degradation complex ERAD (ER-associated degradation) of the endoplasmic reticulum [306]. It behaves in vitro as a disulfide reductase and as a peroxidase [200,306]. 

#### 3.7.9. Selenoprotein-H

Selenoprotein-H is a 23kDa protein which is the only selenoprotein being exclusively localized in the nucleus [307], and it has been shown to protect human fibroblasts against replicative senescence by genome redox maintenance [308,309].

## 4. Bacterial Selenoenzymes

Several selenoenzymes exist in bacteria. Except for selenophosphate synthetase and selenoprotein O which are also found in mammals and catalyze reactions already described in Section 3, bacterial selenoenzymes are mostly involved in anaerobic metabolism [310,311]. They include glycine reductase [312,313,314,315], D-proline reductase [316,317,318], formate dehydrogenase [319,320,321,322,323,324,325] and [NiFeSe]hydrogenase [326,327].

### 4.1. Glycine Reductase

This enzyme catalyzes the reductive deamination of glycine into acetylphosphate [312]:

Glycine + Pi + Thioredoxin dithiol → Acetylphosphate + NH_4_^+^ + Thioredoxin disulfide + H_2_O (6)

It is the first bacterial enzyme that was identified as a selenoenzyme and it may be considered a terminal electron acceptor, acetylphosphate being converted to acetate and ATP by acetate kinase. It enables some anaerobic bacteria to conserve energy via a soluble substrate-level phosphorylation.

Glycine reductase is a trimeric enzyme composed of proteins named A, B. and C. Protein B contains selenocysteine and possibly a pyruvoyl residue. It binds glycine and is responsible for the extrusion of NH_3_. Protein A also contains glycine, which is reduced by thioredoxin, and it provides an intermediate that subunit C uses to produce acetylphosphate. In vitro, the enzyme works with dithiothreitol as a reducing agent, which means that biological reducing cofactors other than thioredoxin cannot be excluded. 

**Figure 8:**
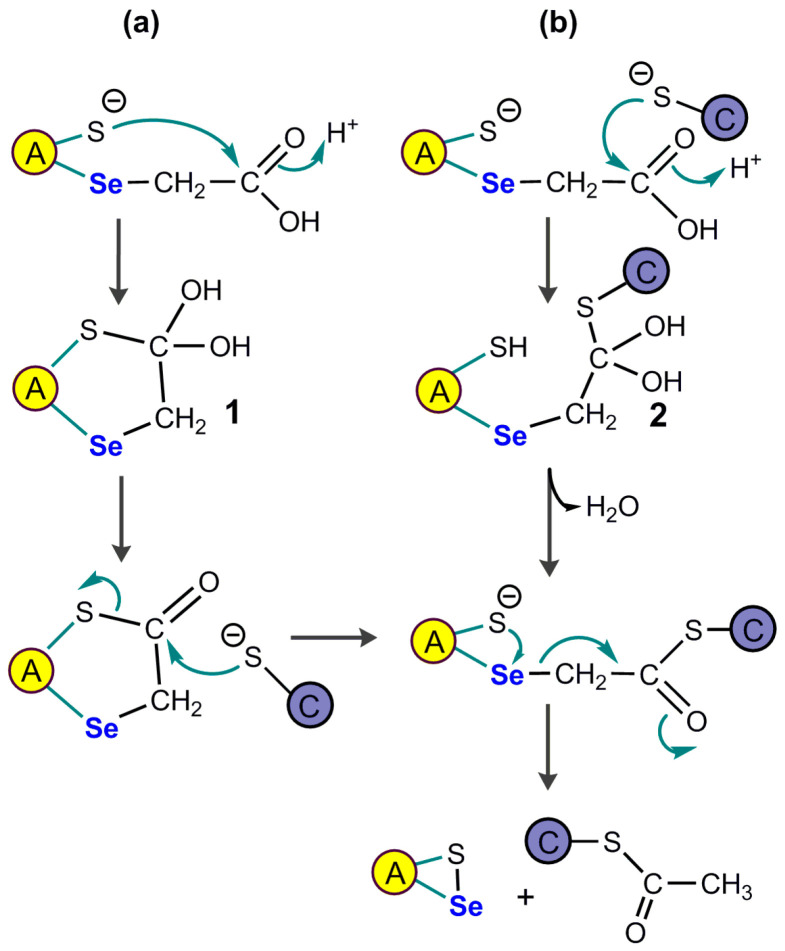
Two possible pathways of formation of the acetyl precursor in glycine reductase. Adapted from Andreesen [314]. The Schiff base formed from glycine and an activated carbonyl group in protein A would react first with a proximal selenocysteine selenolate to yield a carboxymethyl selenoether, whose C2 unit is transformed into an acetyl thioester on protein C, with the formation of a selenenylsulfide bond. Mechanistic options include a speculative keten-like intermediate CH_2_=C=O, and an intramolecular carbonyl hydroxybase 1 in (**a**) or an intermolecular carbonyl hydroxybase 2 in (**b**).

The initial binding of glycine does not involve pyridoxal phosphate but is believed to involve a Schiff base with an activated carbonyl group of the enzyme which has never been clearly identified, due to its instability. It could be a pyruvoyl group, but its formation would require an additional enzyme that would not be within the glycine reductase operon. Some possible transformation steps summarized by Andreesen [314] are shown in Figure 8 in which we underlined that the formation of thioesters would likely require some additional activation step(s) that remain to be identified. In my view, the most interesting reaction here is the thiolate-mediated cleavage of the selenoether bond in the carboxymethylated derivative of selenocysteine, a reaction that had been initially proposed by Arkowitz and Abeles [315,317] without β activation of the distal carboxylate, even though they had in mind that β activation should have been expected. The important point is that the enzyme couples the cleavage of a selenoether carbon-selenium bond to the formation of a selenenylsulfide bond.

### 4.2. D-Proline Reductase

This enzyme catalyzes the reductive cleavage of the D-proline cyclic sidechain into 5-aminopentanoate, which involves breaking of the Cα-N bond [316]:

D-proline + Dithiol → 5-aminopentanoate + Disulfide(7)

This reductive deamination of aminoacid is similar to the reaction catalyzed by glycine reductase, but the existence of an analogous high-energy acyl-enzyme intermediate was excluded by ^18^O_2_ labeling experiments [317], and D-proline reduction is not coupled to substrate-level phosphorylation. The reduction is also NADH-dependent, not NADPH-dependent, which rules out thioredoxin as a biological partner, and is compatible with the use of a coenzyme such as lipoic acid.

D-Proline reductase is a membrane-bound protein composed of 10 subunits. A 46-aminoacid pyruvoyl-containing peptide is covalently attached to each subunit via an ester linkage [318].

**Figure 9:**
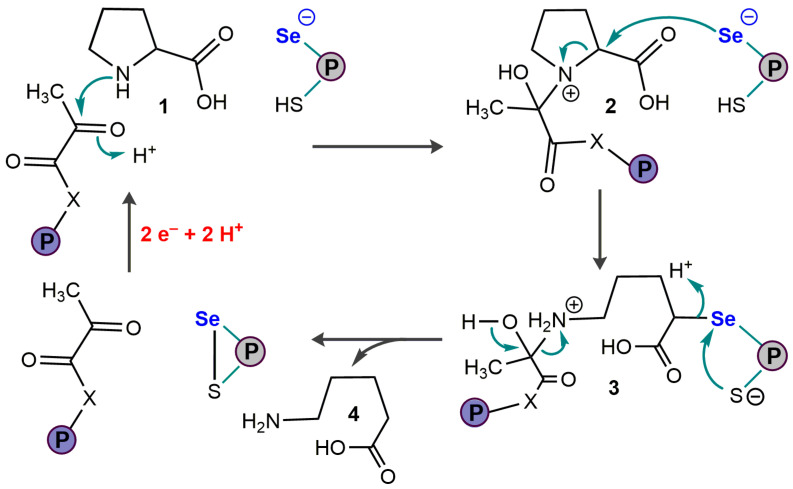
Putative catalytic mechanism of D-proline reductase. Adapted from Kabisch et al. for the enzyme of *Clostridium sticklandii* [316]. SeCys belongs to one protein of the enzyme complex, and pyruvate is covalently bound to another protein. Step 2 → 3 would require an iminium intermediate produced from 2. Step 3–4 involves two independent bond clivages.

The reaction sequence happens on a pyruvoyl-proline covalent adduct, as shown in Figure 9, but the hypothetical mechanism which was proposed [316] relies on two reactions that are not documented as such in organic chemistry. The first one is the nucleophilic attack of the non-activated α carbon of proline, and the second one is the nucleophilic attack of a non-activated selenoether intermediate by a cysteine thiolate, a mechanism that had been initially proposed by the group of Abeles for glycine reductase, as above mentioned [313]. Here again, the important point is that the unusual cleavage of a selenoether carbon-selenium bond is achieved by this selenoenzyme.

### 4.3. Formate Dehydrogenase

Formate dehydrogenases [319,320,321,322,323] catalyze the reversible oxidation of formate to carbon dioxide and play an important role in the global fixation of carbon dioxide, as follows:

HCOOH ⇄ CO_2_ + 2 H^+^ + 2 e^−^ (8)

These enzymes are peripheral membrane proteins located on the cytoplasmic side and have several subunits. Their prosthetic group contains a cis-dithiolene structure which is bound to molybdenum by coordination (structure 11, Figure 1, where two pyranopterin guanine dinucleotides are not represented). The active site also contains an essential selenocysteine residue and a Fe_4_S_4_ cluster. The enzyme from *E. coli* is completely inactivated by iodoacetamide after reduction with formate, and the replacement of selenocysteine with cysteine decreases the activity by more than two orders of magnitude, which confirms the essential function of selenium [320]. Even though they are members of the dimethylsulfoxide reductase family, formate dehydrogenases do not catalyze oxygen-atom transfer reactions.

In its [+IV] oxidation state, molybdenum is pentacoordinate with the four pyranopterine ligands in equatorial positions and a fifth sulfido group in an axial position [324], which gives an approximate square pyramidal geometry. In its [+VI] oxidation state, it adopts a distorted hexacoordinated trigonal prismatic geometry, again with the four pyranopterine ligands plus a fifth ligand which is selenium from the selenocysteine residue and a sixth one which is the sulfido group.

The catalytic mechanism of formate dehydrogenases is still open to debate [323,324,325], but one should underline the existence of functional sulfur homologs in which selenocysteine has been replaced by cysteine. 

**Figure 10:**
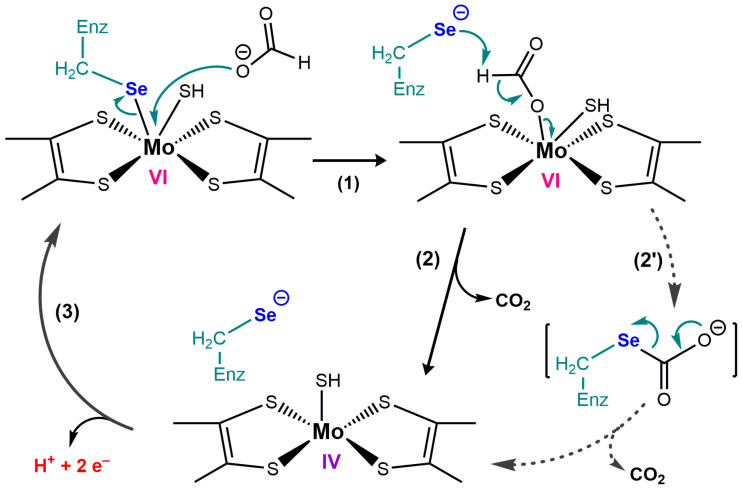
Putative catalytic mechanism of formate dehydrogenase. Formate displaces selenium of the SeCys ligand. It was assumed that the resulting selenolate would assist decarboxylation step (2) as a basic catalyst [323,324], which is unlikely with its low pKa and cannot explain the requirement for selenium. In the alternative of step (2’), the nucleophilic attack of the selenolate would yield a carboxylated SeCys which would decompose to selenolate and CO_2_. The axial ligand of Mo[IV] is sulfide, not selenium.

Figure 10 shows a putative catalytic mechanism that is based on refined structural data as well as functional and theoretical analyses [323,324].

### 4.4. [NiFeSe] Hydrogenases

The structures and functions of [NiFeSe] hydrogenases have been extensively reviewed [326,327]. They are a subclass of [NiFe]-hydrogenases with a selenocysteine residue coordinated to the active site nickel center in place of cysteine. They are exclusively found in sulfate-reducing and methanogenic microorganisms and catalyze the interconversion of hydrogen and protons:

H_2_ ⇄ 2 H^+^ + 2e^−^ (9)

Most of them are heterodimers containing three iron-sulfur clusters in a small subunit and a nickel-iron-containing active site in a large subunit in which there is a selenocysteine ligand. Their prosthetic group was shown as Structure 12 in Figure 1. 

They exhibit highly advantageous properties for applications in water splitting compared with other hydrogenases. They display a high H_2_ evolution rate with marginal inhibition by H_2_ and a high tolerance to O_2_. [NiFeSe]-hydrogenases may therefore be the most efficient catalysts to produce H_2_ by water splitting [291]. An interesting application has been the development of a light-driven full water splitting system with a [NiFeSe]-hydrogenase wired to the water oxidation enzyme photosystem II [328,329].

The mechanism of [NiFeSe] hydrogenases is complex, but the specific advantage of selenocysteine in [NiFeSe] hydrogenase seems to bear in large part on the protection of nickel from irreversible overoxidation by O_2_ [330,331,332]. As discussed in Section 6, one advantage of selenocysteine compared with cysteine may be the better reversibility of its oxidation reactions.

## 5. Is Redox Regulation an Essential Function of Most Mammalian Selenoproteins?

At least in mammals, selenoproteins are all involved in redox transformations. The involvement of GPx4 in redox regulation is supported by the fact that degeneration of GPx4-deficient neuronal cells relies on AIF- and not caspase3-dependent apoptosis [168]. We are dealing here with cell death regulation, however, not with cell function. 

The involvement of TrxR and Msr in the redox regulation of cell function and structure is well established, and the same could be said of the role of deiodinases in metabolic regulations. But the situation is more ambiguous with selenium-glutathione peroxidases because they have major antioxidant properties. There are many arguments that can be used to suggest that they are involved in redox regulation [176,333,334], but one cannot give a detailed description of such regulations. In my view, an antioxidant enzyme could be envisaged as a redox regulator of cell function if its concentration or activity did markedly vary in response to anything else than oxidative stress, but such information is often missing with glutathione peroxidases. Because the information available on GPx3 is insufficient to be conclusive, a good starting point would be to assume that Gpx1 and GPx2 primarily deal with aqueous hydroperoxides, whereas GPx4 should be mainly involved in redox regulations as a phospholipid hydroperoxide reductase. 

As early as 1987, we showed that the intoxication of rats with ethylmorphine, a metabolic source of H_2_O_2_ through cytochrome-P450 demethylases, induced an important increase in hepatic SeGPx after 24 to 48 h [335]. At that time, very little information was known about the mechanisms of transcriptional regulation of enzymes involved in antioxidant protection. One knows today that the promotor of GPx2 contains an ARE sequence (Antioxidant Response Elements) which enables its stimulation in the situation of oxidative stress by dissociation of the Nrf2/Keap1 assembly [111]. But it seems reasonable to assume that this is a purely antioxidant and detoxifying adaptation. GPx4 promoters do not contain any Nrf2 binding site [146], but oxidative stress stimulates the biosynthesis of several selenoproteins (GPx1, GPx4, Trx1, SelS, SelK, and Sps2) by improving the recoding efficiency of UGA/selenocysteine through relocation of the SBP2 elongation factor and the ribosomal protein L30 from the cytoplasm to the nucleus [336]. 

In mouse embryonic fibroblasts, the t-RNA methyltransferase Alkbh8 (mammalian alkylation repair homolog 8) was also shown to markedly induce the expression of several selenoproteins in situations of oxidative stress [337]. Enzymes of the Alkb family are iron-oxoglutarate dioxygenases which perform oxidative demethylation of various DNA and RNA bases and interact with transcription factors. Alkbh8 additionally contains a methyl transferase domain, it is induced in response to activated oxygen species, and it is apparently required for the efficient expression of at least GPx1, GPx2, GPx3, and TrxR1 in this situation [337]. 

A recent study on pure recombinant GPx1, GPx2 and GPx4 shows that they all have significant activities on H_2_O_2_, t-butylhydroperoxide, cumene hydroperoxide, and fatty acid hydroperoxides, but that GPx1 specific activities per selenium are much higher on such substrates [338]. On the other hand, GPx4 is the only one that is efficient on phosphatidylcholine hydroperoxides.

Sometimes, the coexistence of GPx1, GPx2, and GPx3 makes the picture very complex. For example, in the gastrointestinal tract, GPx1 is homogeneously expressed in the intestinal epithelium, whereas GPx2 expression is higher in crypts and mainly in the ileum and cecum [94,339]. As well, GPx3 is also expressed in the gastrointestinal tract while plasma GPx3 binds to the basement membranes of intestinal epithelial cells [143].

Hydrogen peroxide H_2_O_2_ is produced by the stimulation of many hormone receptors, and it is believed to serve as an intracellular messenger by directly or indirectly oxidizing cysteine residues of target proteins [340,341]. Multiple H_2_O_2_ primary or secondary sensors contribute to the redox regulation of transcription factors which include among others AP-1, Nrf2, and NF-kB, and this complex network regulates major cellular events such as proliferation, differentiation, and apoptosis [342,343]. Thus, GPx1 and perhaps GPx2 might be involved in redox regulation by controlling H_2_O_2_ concentrations in space and time, but this is virtually impossible to prove without detailed information on the flux of H_2_O_2_ production by an activated enzyme such as NADPH oxidases as well as on their variations of activity. H_2_O_2_ may also have multiple time-dependent effects, for example, it acts as a biphasic modulator of NF-kB activation by other agents, not only as a direct activator [344,345].

At any rate, peroxiredoxins (Prx)—which are not selenoproteins may be more dedicated to H_2_O_2_ signaling than GPx1 or GPx2, and they use the small reducing pool of thioredoxin under the control of TrxR, not the pool of GSH which is generally in large excess. Given their reduction by the Trx/TrxR system, they are under the control of selenoenzymes, however.

One may have overlooked the importance of glutathione peroxidases in the control of organic hydroperoxide concentrations. Potential targets of such regulatory effects include at least lipoxygenases [120,121,122] and cyclooxygenases [346] whose activities require an initiation step induced by preformed organic hydroperoxides, and phosphatases, especially protein tyrosine phosphatase [347]. The latter invariably contains an essential cysteine thiolate in their active site which can be oxidized to specific sulfenic, sulfenylamide, or disulfides. The direct oxidation of such active site cysteines by H_2_O_2_ is very slow [347], but it was shown that physiological concentrations of bicarbonate which reacts with H_2_O_2_ to produce peroxymonocarbonate, facilitate the H_2_O_2_-mediated inactivation of protein tyrosine phosphatase 1B [348]. The observation that GPx1 or GPx4 overexpression can inhibit the activation of NFkB [161,349] would also fit with oxidative inactivation of a specific phosphatase. But with GPx1 as well as with GPx4, we do not know which hydroperoxides are directly involved and which target protein is affected.

Binding sites for transcription factors distinct from Nrf2 have been identified on GPx1 as well as GPx4 genes [176], but little is known about corresponding regulatory functions.

Finally, if the activity of a given SeGPx was not only upregulated for antioxidant protection but at least transiently downregulated for other purposes, for example by means of phosphorylation or mixed disulfide formation, it would certainly qualify as a redox regulator. We already mentioned that GPx4 might be under redox control of the adaptor protein 14-3-3e [180]. In cell culture, artificial inhibition of GPx1—and perhaps GPx2 and GPx4—by mercaptosuccinate markedly increases Ca^2+^-mediated activation of monocyte 5-lipoxygenase [121]. At least one biological example of GPx4 down-regulation is associated with intestinal trans-epithelial neutrophil migration. This process is controlled by 12-lipoxygenase which produces the eicosanoid hepoxilin A3 and establishes a chemotactic gradient that guides PMN across the epithelial surface. Using Salmonella typhimurium to induce this process, it was found that the bacteria-induced apical secretion of hepoxilin A3 by decreasing the expression of GPx4, which in turn led to an increase in 12-lipoxygenase activity [350].

Mammalian tyrosine kinases c-Abl and Arg, which are devoid of receptors and play an important role in apoptosis induced by oxidative stress, form constitutive complexes with GPx1, thereby protecting the enzyme at non-lethal doses of H_2_O_2_, whereas lethal doses of H_2_O_2_ destroy such complexes and induce apoptosis [351]. This suggests that some growth factors may regulate SeGPx activities. At least one study showed that VEGFB strongly induced the expression of GPx1 and more modestly that of GPx5 in murine retinal cells [352].

Overall, we tentatively conclude that SeGPx is involved in major redox regulatory networks but may be more as local “kinetic buffers” of hydroperoxide concentrations than as dedicated transducers of redox signals such as protein regulators under the control of the Trx/TrxR system.

## 6. Advantages and Constraints Associated with the Choice of Selenocysteine at the Active Site of Selenoenzymes

Given the similarities between sulfur and selenium properties, a question that the biochemist cannot bypass is that of the expensive selection of selenium rather than that of sulfur [353,354,355,356,357]. Under its thiolate form, sulfur is indeed a good nucleophile and a good reductant. Contrary to vertebrates, non-vertebrate animals, higher plants, and fungi produce many protein homologs of our selenoproteins, in which selenocysteine is replaced by cysteine, but their peroxidase activities have been studied in detail only for non-vertebrate CysGPx. Such non-vertebrate CysGPx is not simple cysteine homologues as suggested by their name, because they contain an active site cysteine (peroxidatic Cp) in place of selenocysteine, but also a second cysteine (resolving Cr) which is used to form a disulfide from a putative cysteine sulfenic acid intermediate (reviewed in 188). This disulfide is then recycled by thioredoxin (or other redoxins), not by GSH. Some of these CysGPx/thioredoxin peroxidases are surprisingly efficient to reduce hydroperoxides, although not as efficient as the selenium-dependent GSH peroxidases, having typically second-order rate constants for the hydroperoxide reduction step which are at least two orders of magnitude smaller [188].

The case of peroxiredoxins already cited and which are very efficient peroxidases on H_2_O_2_ is another interesting example, even though such enzymes are not selenoprotein homologs. 

If one inspects the key steps of the catalytic cycles of selenoenzymes that are best understood (see Figure 5), one would be tempted to say that a cysteine thiol group that would be activated to thiolate should be able to do the job. In selenoenzymes, the initial step is a nucleophilic attack by the selenolate group, and the final step is its restitution as leaving group. Conceivably, a thiolate group could replace the selenolate since the formation of a cysteine thiolate is generally observed in enzymes bearing a catalytic cysteine. Examples include glyceraldehyde-phosphate-dehydrogenase and disulfide reductases such as GSSG reductase, lipoamide dehydrogenases, thioredoxins, and glutaredoxins. 

It is often underlined that the second-order rate constant of the active site selenolate of GPx1 with H_2_O_2_ is close to 5.10^7^ M^−1^.s^−1^, whereas that of a non-enzymatic selenolate group is several orders of magnitude smaller. This would suggest that the protein environment of the active site selenocysteine is doing much of the job, and recent DFT calculations on a modelized H_2_O_2_/SeGPx or H_2_O_2_/SGPx interaction would support this concept [358,359]. In these model studies, it was found that the presence of one water molecule in the active site triggered a concerted deprotonation/reprotonation in which the proton dissociated from the selenol or thiol group was transferred by the bridging water to one oxygen of the peroxide bond, ensuring an optimal concerted nucleophilic attack of the peroxide bond by the selenolate or thiolate. This theoretical study does not explain however why the exchange of selenium for sulfur at the active site of GPx1 results in a 1000-fold decrease in GPx activity [360]. Similarly, a marked GPx activity often results from the exchange of native sulfur for selenium in cysteine-dependent enzymes which are not GPx and have no measurable GPx activities [361,362]. Such enzymes include subtilisin [363,364], GST [365], glutaredoxin [366,367], and even glyceraldehyde-3-phosphate dehydrogenase [368]. 

But one should admit that the example of peroxiredoxins whose second-order rate constants with H_2_O_2_ are close to 10^7^ M^−1^.s^−1^ invalidates the concept of an absolute requirement for selenium in fast peroxide bond reduction. 

If we compare the properties of oxygen, sulfur, and selenium, there is a drastic change in properties in going from oxygen to sulfur, whereas the changes are more modest in going from sulfur to selenium. The covalent radii change more between the first and the second rows than between succeeding rows, which is illustrated by the abrupt change in electronegativity between oxygen and sulfur, much more modest from sulfur to selenium. In water, the nucleophilic selenolate form will predominate at neutral pH, which is not the case of the thiolate form, but given the ease of pKa lowering of cysteine thiols in enzyme active sites, a relevant comparison should bear on selenolate versus thiolate.

With its 3d-orbitals, a selenolate group is a softer nucleophile than a thiolate group, and this could be a major advantage for interaction with iodine in deiodinases, but to which extent this could facilitate the nucleophilic attack of a peroxide bond is not obvious. Perhaps a selenium-peroxide transition state of non-conventional geometry should be envisaged. Similar geometries of sulfur and selenium transition states have always been used in theoretical comparisons.

Another difference between thiols and selenols bears on their protonated form RSH and RSeH. Many years ago, Huber and Criddle [369] showed that the selenol form RSeH of selenocysteine had significant nucleophilic properties in water, which was not the case for the thiol form RSH of cysteine. Again, this is likely to come from filled 3d orbitals in selenium [empty in sulfur] and from the fact that with very similar covalent radii of sulfur and selenium, the electron density in the 3d and/or 4p orbitals of selenium should be much higher than that of the sulfur outer shell.

Many potentially discriminating properties have been extensively discussed in two reviews by Hondal and coworkers [356,357]. They confirm that selenolates are more nucleophilic than thiolates, and that selenides are more nucleophilic than sulfides. As well, selenium nucleophilicity may not be the main discriminating factor because electrophilic groups such as diselenides, selenenic acids, and selininic acids are also much better electrophiles than their sulfur analogs [356]. The reduction rate of seleninic acids by thiols is at least 10^6^ times faster than the reduction rate of sulfinic acids by thiols [356] and this may be where the discrimination is most obvious. As underlined by Reich and Hondal [356], it would explain an observation that we had made on glutathione peroxidase [360]. By expressing the first sulfur/cysteine mutant (S-GPx) of GPx1 by directed mutagenesis, we had not only observed an enzyme activity that was decreased by three orders of magnitude, but also a fast auto-inactivation whose rate increased with the (hydroperoxide)/(GSH) ratio, and this had led us to envisage an irreversible mechanism of sulfur overoxidation which would not occur with selenium. At that time, our conclusion was that selenium solved a problem of irreversible oxidation that might induce sulfur loss by β-elimination, but we had no structural data to show. A specific method of immunodetection of dehydroalanine residues was developed only much later [370]. It was used to show that Sec in red blood cell GPx1 was increasingly replaced with dehydroalanine during blood storage.

It was also shown on the GPx4 cysteine mutant of *Schistosoma mansoni* that the active-site cysteine was oxidized to Cys-sulfonate, which did explain its inactivation [371]. Much more recently, it was shown that rat GPx4 was built to avoid irreversible overoxidation of selenium in the situation of GSH deficit thanks to the attack of the selenenic acid Enz-SeOH by a nucleophilic nitrogen from the polypeptide backbone [372]. The protective Se-N bond is broken by GSH when its physiological concentration is re-established, and the system is ready for new catalytic cycles.

Sulfur mutants of other selenocysteine-containing enzymes have been studied, including the [NiFeSe] hydrogenases discussed in Section 4.4, and as a rule, they were found to undergo oxidative inactivation which was not observed with the selenocysteine-containing enzymes [357]. Overall, it may be concluded that the catalytic advantage of selenium compared with sulfur relies in large part on a unique combination of faster kinetics and much better reversibility of its oxidation reactions.

## *7.* Conclusions

The extensive investigations and multiple discoveries of the last four decades have been very exciting, and a few points deserve to be underlined:

- The co-translational incorporation of selenium in selenoproteins is probably unique among the elements situated below period 3 of the periodic table, and one of the major advantages of selenium compared with sulfur should be the better reversibility of its oxidation reactions in biological conditions.
- Selenoproteins are mostly involved in anaerobic metabolism in bacteria, whereas they are involved in antioxidant protection, protein repair, redox signaling, and regulation of cell proliferation/cell death, and energetic metabolism in mammals.
- Many functions of mammalian selenoproteins, especially those which are anti-inflammatory, anti-apoptotic, or anti-ferroptotic, or which interfere with energetic metabolism, require regulation in space and time. Specifically, most mammalian selenoenzymes should not only be regulating but also regulated, although, in this area, much information is probably still missing.
- With the recent development of new techniques of selenocysteine insertion into protein sequences, we can expect that new discoveries will shed light on the hidden face of the moon.

## Data Availability

No original data available with this review article.

## Abbreviations

AIF, apoptosis inducing factor; Alkbh, mammalian alkylation repair homolog; Alox15 gene, gene encoding an arachidonate 15-lipoxygenase; AP-1, activator protein 1; ASK1, apoptosis signal kinase 1; CAMKII, Ca^2+^ calmodulin-dependent kinase; COX2, Type 2 cyclooxygenase; DIO, deiodinase; DUOX, dual oxidase; EFsec, selenocysteine dedicated elongation factor; EMT, epithelial-mesenchymal transition; GLS2, liver-type glutaminase; GPx, glutathione peroxidase; GR, glutathione reductase; HCV, hepatitis C virus; HIF, hypoxia inducible factor; Keap1, Kelch associated protein 1; LOX, Lipoxygenase; LPS, lipopolysaccharide; MAPK, mitogen-activated protein kinase; MICAL, molecules interacting with CasL; MsrB1, methionine sulfoxide reductase B1; NFκB, nuclear factor kB; Nox, NADPH oxidase; Nrf2, nuclear factor erythroid-2-related factor; NQQ1, NAD[P)H: Quinone oxidoreductase 1; PDI, protein disulfide isomerase; Prx, peroxiredoxin; PTEN, phosphatase and tensin homolog; SBP2, SECIS-binding protein 2; SecTRAPs, selenium compromised thioredoxin reductase-derived apoptotic proteins; SECYS, selenocysteine insertion sequence; Selenoprotein P, selenoprotein P; Sephs2, selenophosphate synthetase 2; TGS1, trimethylguanosine synthase 1; TNFα, tumor necrosis factor a; TPO, thyroid peroxidase; Trx, thioredoxin; TrxR, thioredoxin reductase; T4, thyroxine; T3 triiodothyronine; ZEB1, zinc-finger E-box binding protein 1.
